# Negative regulation of EGFR signalling by the human folliculin tumour suppressor protein

**DOI:** 10.1038/ncomms15866

**Published:** 2017-06-28

**Authors:** Laura A. Laviolette, Julien Mermoud, Isabel A. Calvo, Nicholas Olson, Myriam Boukhali, Ortrud K. Steinlein, Elisabeth Roider, Elke C. Sattler, Dachuan Huang, Bin Tean Teh, Mo Motamedi, Wilhelm Haas, Othon Iliopoulos

**Affiliations:** 1 Center for Cancer Research, Massachusetts General Hospital Cancer Center and Harvard Medical School, Boston, Massachusetts 02139, USA; 2 Institute of Human Genetics, University Hospital Munich, University of Munich, Munich 80336, Germany; 3 Department of Dermatology and Allergology, University Hospital, Ludwig Maximilian University Munich, Munich D-80337, Germany; 4 Laboratory of Cancer Epigenome, Division of Medical Sciences, National Cancer Centre Singapore, Singapore 169610, Singapore; 5 Cancer and Stem Cell Biology Program, Duke-NUS Medical School, Singapore 169610, Singapore; 6 Division of Hematology-Oncology, Department of Medicine, Massachusetts General Hospital, Boston, Massachusetts 02114, USA

## Abstract

Germline mutations in the Folliculin (*FLCN*) tumour suppressor gene result in fibrofolliculomas, lung cysts and renal cancers, but the precise mechanisms of tumour suppression by FLCN remain elusive. Here we identify Rab7A, a small GTPase important for endocytic trafficking, as a novel FLCN interacting protein and demonstrate that FLCN acts as a Rab7A GTPase-activating protein. FLCN^−/−^ cells display slower trafficking of epidermal growth factor receptors (EGFR) from early to late endosomes and enhanced activation of EGFR signalling upon ligand stimulation. Reintroduction of wild-type FLCN, but not tumour-associated FLCN mutants, suppresses EGFR signalling in a Rab7A-dependent manner. EGFR signalling is elevated in FLCN^−/−^ tumours and the EGFR inhibitor afatinib suppresses the growth of human FLCN^−/−^ cells as tumour xenografts. The functional interaction between FLCN and Rab7A appears conserved across species. Our work highlights a mechanism explaining, at least in part, the tumour suppressor function of FLCN.

## Abstract (web summary)

Folliculin is a known tumour suppressor but the molecular mechanisms behind this function are unclear. Here the authors show that Folliculin regulates EGFR signalling by modulating its Rab7a-dependent trafficking.

Individuals with Birt–Hogg–Dubé (BHD) disease are at an increased risk of developing renal cell cancers, benign skin lesions called fibrofolliculomas, and lung cysts^1^,^2^,^3^. BHD is a rare disease of unclear incidence and of high penetrance^4^, caused by germline mutations in the folliculin (*FLCN*) gene and most mutations predict for a truncated form of the protein missing the C terminus^2^,^3^,^5^. However, specific missense and in-frame deletions in FLCN have been observed in individuals with BHD disease, such as K508R and dF157 (ref. 5). FLCN encodes an evolutionarily conserved 64 kDa phospho-protein that is ubiquitously expressed in adult and embryonic tissues and is localized to both the nucleus and the cytoplasm^6^,^7^,^8^,^9^. Somatic inactivation or loss of the wild-type (WT) *FLCN* allele is observed in the renal tumours of patients with BHD disease and in sporadic renal cell carcinomas, suggesting that FLCN acts as a tumour suppressor^9^,^10^.

Very little is known about the precise mechanisms of tumour suppression by human FLCN. Previous studies demonstrated that FLCN interacts with folliculin-interacting proteins 1 and 2 (FNIP1 and FNIP2), the Rag GTPases A and C/D, GABA(A) receptor-associated protein (GABARAP), and plakophilin-4 (refs 11, 12, 13, 14, 15, 16, 17, 18). Although there has been strong evidence indicating a functional interaction between FLCN and mTORC1, the complex biochemical details of this functional interaction are currently under investigation. Mammalian target of rapamycin (mTOR) is a conserved serine/threonine kinase that is part of the multiprotein mTOR complex 1 (mTORC1); the latter couples growth factors, and amino acid and energy availability to cell growth and autophagy and its activity is upregulated in many human cancers^19^,^20^. It has been initially reported that FLCN–FNIP1/2 interactions occur in the cytoplasm as part of a larger complex with the γ-subunit of AMPK, indicating that FLCN may be involved in nutrient sensing and cellular metabolism through the AMPK-mTOR signalling pathway^12^. Subsequently, FLCN was shown to be required for the recruitment and activation of mTORC1 in response to amino acids through its interaction with Rag GTPases at the lysosome^17^,^18^.

The C terminus of FLCN (amino acids 341–579) was crystalized and found to contain a DENN domain by structural analysis^21^. DENN domain proteins function as guanine nucleotide exchange factors (GEFs) that activate Rab GTPases by mediating the exchange of GDP for GTP^22^. The Rab family of small GTPases coordinate critical aspects of eukaryotic membrane trafficking, including vesicle budding, uncoating, motility and fusion, and is a large family consisting of over 60 members^23^. Rab GTPases cycle between GTP-bound and GDP-bound forms. GEF domain containing proteins promote the transition from the GDP-bound and inactive form to GTP-bound and active form. TBC (Tre-2/Bub2/Cdc16) domain proteins act as GTPase activating proteins (GAPs) promoting GTP hydrolysis and accelerate transition of GTPases to the ‘inactive’ GDP-bound form^24^. Consistent with the crystal structure data and putative role of FLCN as a GEF protein, FLCN was shown to interact with Rag GTPases at the lysosome^17^,^18^. In one study, FLCN possessed GTPase-activating protein (GAP) activity for Rag C/D^18^, while another study suggested that FLCN may act as a GEF for RagA^17^. In these studies, FLCN was required for the recruitment and activation of mTORC1 in response to amino acids. The model proposed by these studies predicts that loss-of-FLCN function would lead to suppression of mTORC1 function; such a model contradicts the role of FLCN as a tumour suppressor. Previous experiments performed *in vitro* versus *in vivo* have yielded conflicting results about FLCN’s ability to inhibit or activate mTORC1 (refs 12, 17, 18, 25, 26, 27).

To gain insight into the cellular function of FLCN, we isolated FLCN protein complexes and identified a novel interaction between FLCN and the Rab GTPase, Rab7A. Our results suggest that FLCN regulates Rab7A’s GTPase activity by acting as a Rab7A GAP. Rab7A functions in the endosomal recycling and lysosomal degradation of epidermal growth factor receptor (EGFR), two key processes that regulate EGFR stability, expression and signalling^28^,^29^,^30^. EGFR is a cell surface receptor tyrosine kinase that is often overexpressed or mutated in human cancers, resulting in increased proliferation, migration and angiogenesis^31^. Importantly, we found that FLCN^−/−^ cells have increased EGFR signalling upon EGF ligand activation (phosphorylated EGFR (pEGFR), pERK and pS6) and that stable expression of exogenous Rab7A in the FLCN^−/−^ cells decreased EGFR signalling, demonstrating that Rab7A is sufficient to rescue the EGFR signalling phenotype in these cells. In addition, FLCN^−/−^ cells display slower endosomal trafficking of EGFR from early endosomes to late endosomes and from late endosomes to lysosomes, compared to FLCN-replete cells. Taken together, our data suggest that the interaction between FLCN and Rab7A is important for EGFR cellular trafficking and that misregulation of Rab7A activity due to FLCN loss results in slower EGFR trafficking and increased EGFR signalling.

## Results

### FLCN functions as a Rab7A GTPase-activating protein

**Figure 1:**
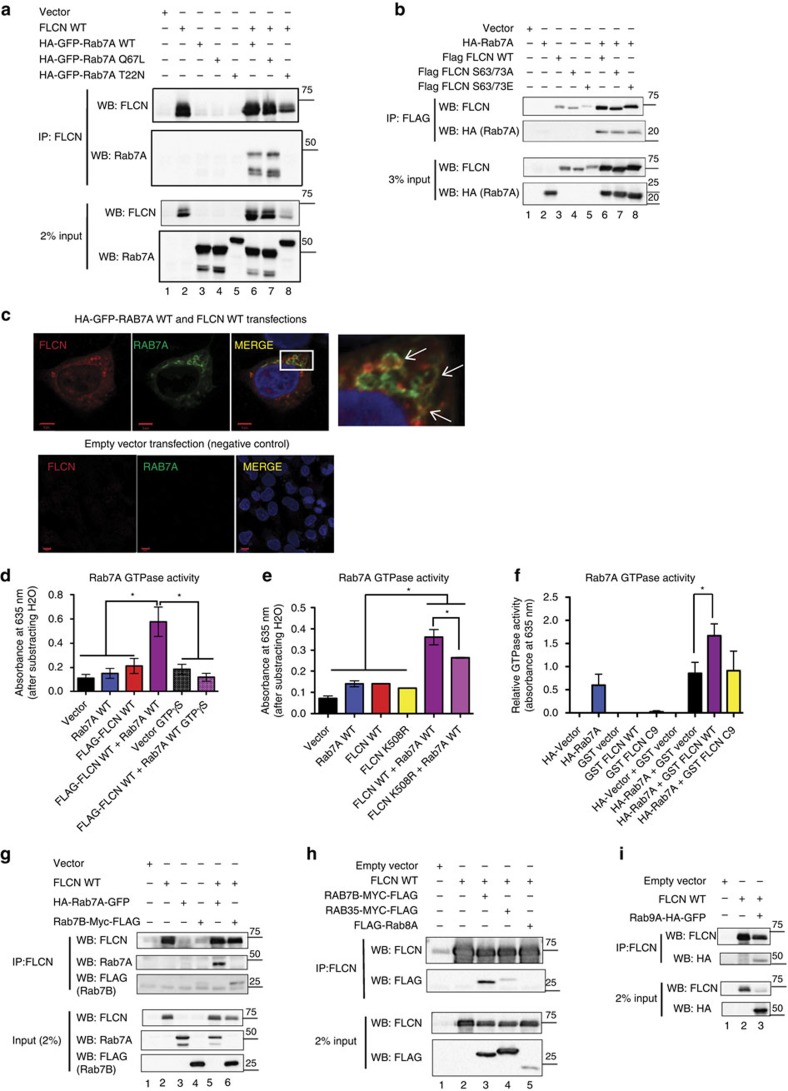
FLCN is a GAP for Rab7A. (**a**) U2OS cells were transiently transfected with empty vector, FLCN WT, Rab7A WT, Rab7A Q67L, Rab7A T22N or combinations, as indicated. Co-purified complexes were detected by immunoblotting with antibodies recognizing FLCN or Rab7A, as indicated. (**b**) 293T cells were transiently transfected with an empty vector, FLAG-FLCN WT, FLAG FLCN S62/73A, FLAG FLCN S62/73E, HA-Rab7A WT or combinations, as indicated. Co-purified complexes were detected by immunoblotting with antibodies recognizing FLCN or HA (Rab7A), as indicated. (**c**) U2OS cells were transfected with empty vector, or FLCN WT and HA-GFP-Rab7A. Co-localization was identified by confocal microscopy (yellow, indicated by arrows in the insert corresponding to the region outlined by the white box). Scale bars, 5 μm (upper panel) and 10 μm (lower panel). (**d**) HA-Rab7A and FLAG-tagged FLCN WT proteins were purified from transfected 293T cells with anti-HA (Rab7A) or anti-FLAG antibodies. The amount of inorganic phosphate released due to GTPase activity was measured by GTPase colorimetric assay kit. GTPase activity was quantified in four independent experiments, and data are represented as mean±s.e.m. Significance (*) was conferred at *P*<0.05, ANOVA and Tukey’s Multiple Comparison post-tests. (**e**) Same as in **d**, except FLCN WT and FLCN K508R (untagged) were immunoprecipitated with an anti-FLCN antibody from the lysates of transfected 293T cells. The data are represented as mean±s.e.m. and were collected in two independent experiments. * indicates statistical significance, *P*<0.05, ANOVA and Tukey’s Multiple Comparison post-tests. (**f**) GST-FLCN WT or GST-FLCN C9 mutant proteins were incubated with HA-Rab7A, purified from transfected 293T cells. The GAP activity was measured as in **d**,**e**. Data are represented as mean±s.e.m., *n*=5. * indicates statistical significance, Two-tailed paired *t*-test, *P*=0.0037. (**g**) U2OS cells were transiently transfected with an empty vector, FLCN WT, Rab7A WT, Rab7B or combinations, as indicated. Co-purified complexes were identified by immunoprecipitation followed by immunoblot, as indicated. (**h**) Same as in **g**, except U2OS cells were transiently transfected with Rab7B, Rab35 or Rab8A, as indicated. (**i**) Same as in **g**, except U2OS cells were transiently transfected with Rab9A.

In order to gain insight into the cellular functions of FLCN, we purified protein complexes from the FLCN-deficient UOK257 cell line and UOK257 cells stably expressing Flag-tagged WT FLCN. To increase the depth of FLCN interactome recovery, we fractionated cells into nuclear, cytoplasmic and cell membrane fractions, purified FLCN protein complexes in each fraction, and analysed the fractions by mass spectrometry. Our mass spectrometry analysis revealed several FLCN interacting proteins, including the known interactors FNIP1, FNIP2 and GABARAP (Supplementary Data 1). Because the C terminus of FLCN was previously shown to have structural homology to the DENND1B protein and GEF activity towards Rab35 (ref. 21), we were particularly interested in finding novel interactions between FLCN and Rab GTPases. We found several Rab proteins that interact with FLCN, but the small GTPase, Rab7A, had the highest spectral count in the membrane fraction (active Rab GTPases are localized to endocytic vesicles^23^) of FLCN WT cells (and no spectral counts in the FLCN^−/−^ membrane fraction, Supplementary Data 1). The novel FLCN–Rab7A interaction was confirmed by co-immunoprecipitation (IP) and co-localization by immunofluorescence (IF) in U2OS cells (Fig. 1a–c). U2OS cells (which express low levels of endogenous FLCN) were co-transfected with FLCN WT and HA-GFP-tagged wild-type Rab7A (WT), constitutively active (CA) Rab7A Q67L mutant or dominant negative (DN) Rab7A T22N mutant^32^ (Fig. 1a). Notably, IP of FLCN in U2OS cells demonstrated preferential binding of FLCN to the Rab7A WT protein (Fig. 1a, lane 6) and the GTP-bound CA Q67L mutant (Fig. 1a, lane 7), but no binding to the GDP-bound DN Rab7A T22N mutant (Fig. 1a, lane 8). The Rab7A T22N mutant displays an apparent molecular weight slightly higher than the WT or CA species, most likely due to the longer linker in the vector between the HA-EGFP tag and Rab7A. Although we favour the hypothesis that Rab7A T22N does not bind to FLCN due to GDP load, we cannot exclude the possibility that the presence of post-translational modifications may be responsible for the lack of binding to FLCN. We interpreted the data as suggesting that the preferential binding of FLCN to Rab7A WT and Rab7A CA underscores the possibility that FLCN acts as a Rab7A GAP. Rab7A and FLCN were not present in the control IP with IgG antibody, suggesting that the interaction between FLCN and Rab7A (both the WT and CA forms) is specific (Supplementary Fig. 1). The co-transfection of FLCN and Rab7A T22N in cells resulted consistently in lower expression of FLCN (Fig. 1a lane 8, see FLCN input) suggesting a putative feedback loop between Rab7A and FLCN. The phosphorylation of FLCN on S62 and S73 was previously shown to be important for cell cycle regulation^33^, but did not affect FLCN’s ability to bind Rab7A. Both the phosphomimetic mutant form of FLCN (S62/73E) and the phosphoinactive mutant form of FLCN (S62/73A) bound Rab7A at similar levels to WT FLCN (Fig. 1b). Similarly, FLCN and Rab7A were shown to co-localize in vesicular structures in the cytoplasm of transfected U2OS cells, as indicated by the arrows (co-localization is identified by the yellow areas (arrows), Fig. 1c). Using several truncated mutant forms of the FLCN WT protein, we found that the Rab7A-binding domain of FLCN is contained within amino acids 450–579 (Supplementary Fig. 2). This C-terminal region of the FLCN protein is often lost in BHD patients due to truncating mutations, and is also part of the putative DENN domain of FLCN (amino acids 340–579).

We hypothesized that FLCN acts as a GAP for Rab7A, because, as presented above, it appeared to bind preferentially to the GTP-bound CA Q67L mutant (Fig. 1a, lane 7), but not to the GDP-bound DN Rab7A T22N mutant (Fig. 1a, lane 8). To further characterize FLCN’s activity as a Rab7A GAP, we used a commercially available GTPase assay kit (Innova Biosciences) to measure the amount of inorganic phosphate (Pi) produced by Rab7A’s enzymatic hydrolysis of GTP. The colorimetric assay utilizes the PiColorLock Gold reagent to detect Pi when read at a wavelength of 635 nm. Rab7A WT, FLCN WT or the tumour-associated FLCN K508R mutant were purified from transfected 293T cells. Immunoprecipitates (using the same antibodies and beads) from 293T cells transfected with Vector (pCDNA3.1) were used as negative controls. A non-hydrolysable form of GTP (GTP**γ**S) was also used as a negative control. FLCN WT protein or Rab7A WT protein alone had GTP hydrolysis levels similar to our negative controls (vector control (beads and antibody and containing no purified protein) and the GTP**γ**S) (Fig. 1d), demonstrating that individually, these proteins hydrolyse very little GTP. When FLCN WT protein was combined with Rab7A WT protein, there was a significant increase in GTP hydrolysis (∼5-fold increase over the vector (beads) control; Fig. 1d). The ability of FLCN WT to increase the enzymatic activity of Rab7A and increase GTP hydrolysis indicates that FLCN functions as a Rab7A GAP (Fig. 1d). A missense mutant form of FLCN associated with kidney tumorigenesis in BHD families, FLCN K508R, was used in the GTPase assay because, although mutated, the protein is stably expressed at levels similar to FLCN WT^3^,^5^,^33^. Interestingly, the tumour-associated mutant K508R displayed decreased Rab7A GAP activity and was not as effective as FLCN WT at stimulating GTP hydrolysis (Fig. 1e). Confirmation of the presence of FLCN WT and mutant proteins in the immunoprecipitates used for the GAP assay is provided in Supplementary Information (Supplementary Fig. 3). These data suggest that the tumour suppressor function of FLCN may be, at least in part, linked to its ability to act as a GAP protein. To ensure that the GAP activity of FLCN is not due to co-purification of other mammalian proteins, we GST-purified FLCN WT protein, or a tumour-associated mutant form of FLCN (FLCN C9) from bacteria and tested their ability to hydrolyse GTP when combined with Rab7A. FLCN WT significantly increased the GTPase activity of Rab7A compared to the GST vector alone (Fig. 1f), and there was a trend towards decreased GTPase activity of the tumour-associated mutant form of FLCN (FLCN C9) (Fig. 1f).

Because of our mass spectrometry results (Supplementary data 1) and FLCN’s putative DENN domain in the C terminus, we asked whether FLCN interacts with other Rab GTPases. U2OS cells were co-transfected with FLCN and several Rab GTPases, Rab7B (Fig. 1g lane 6 and Fig. 1h, lane 3), Rab35 (Fig. 1h, lane 4), Rab8A (Fig. 1h, lane 5) or Rab9A (Fig. 1i, lane 3). All tested Rabs co-immunoprecipiated with FLCN except Rab8A (Fig. 1h, lane 5). The FLCN-binding affinity varied for each of the Rabs, with Rab7A (Fig. 1a (lane 6), Fig. 1b (lane 6) and Fig. 1g (lane 5)) and Rab7B (Fig. 1g (lane 6) and Fig. 1h (lane 3)) having the strongest interaction with FLCN.

### FLCN^−/−^ cells have delayed endocytic trafficking of EGFR

**Figure 2:**
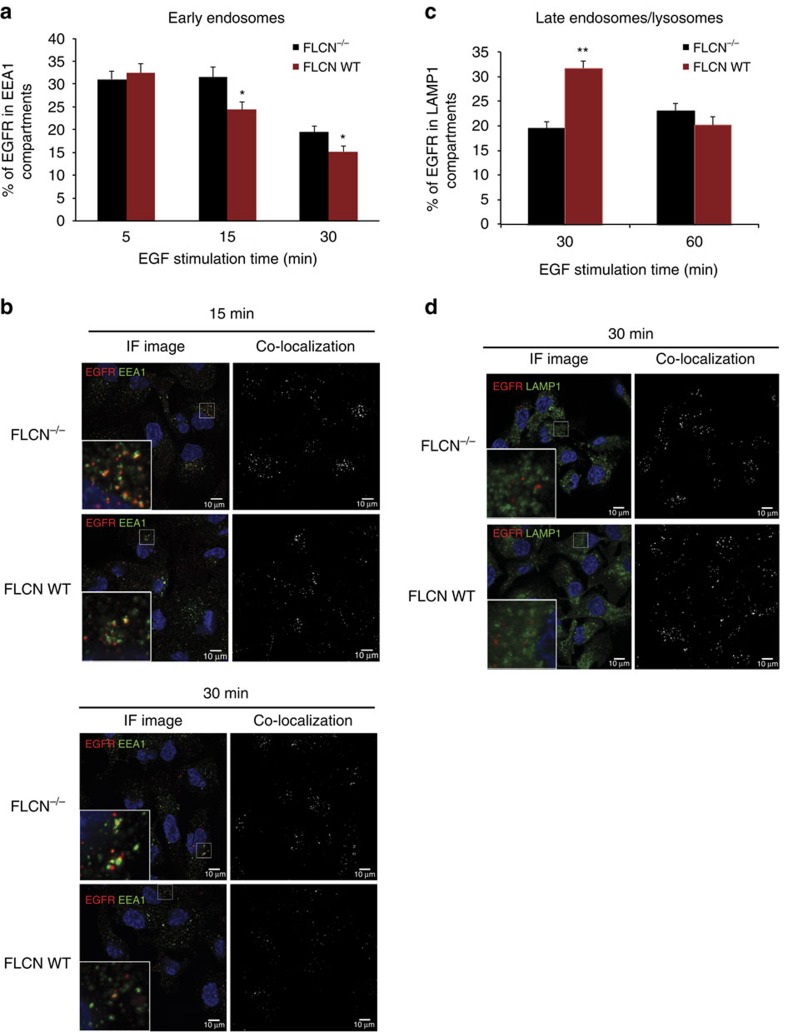
Loss of FLCN results in slower endocytic trafficking of EGFR. (**a**) FLCN-deficient UOK257 cells (FLCN^−/−^) and wild-type FLCN-replete cells (FLCN WT) were starved for 2 h in RPMI 1640 (containing no growth factors and amino acids), then stimulated with EGF (1 μg ml^−1^), and fixed for immunofluorescence and confocal microscopy. The percentage of EGFR co-localizing with EEA1 for each time point was quantified from 18 pictures (5–10 cells/picture) taken from three independent experiments, and is presented as the mean±s.e.m. * indicates significance, *t*-test, *P*<0.05. (**b**) Squares on the left show confocal microscopy images of immunofluorescence with anti-FLCN and anti-EEA1 antibody (IF image). Squares on the right present the foci of co-localization as identified by ImageJ analysis (co-localization). Upper panel images were taken at 15 min and lower panel images taken at 30 min, as indicated. (**c**) FLCN-deficient UOK257 cells (FLCN^−/−^) and wild-type FLCN-replete cells (FLCN WT) were starved for 2 h in RPMI 1640 (containing no growth factors and amino acids) in the presence of chloroquine diphosphate salt (100 μM), then stimulated with EGF (1 μg ml^−1^), and fixed for immunofluorescence and confocal microscopy. The percentage of EGFR co-localizing with LAMP1 for each time point was quantified from 18 pictures taken from three independent experiments and is presented as the mean±s.e.m. ** indicates significance, *t*-test, *P*<0.0005. (**d**) Squares on the left show confocal microscopy images of immunofluorescence with anti-FLCN and anti-LAMP1 antibody (IF image). Squares on the right present the foci of co-localization as identified by ImageJ analysis (co-localization).

Rab7A is a small GTPase that is located in late endosomes, lysosomes and autophagosomes and functions in endocytic trafficking of cargo proteins, including EGFR^23^. Following EGF ligand binding and EGFR internalization, Rab7A plays an important role in both recycling EGFR to the cell surface and degrading EGFR in lysosomes^28^,^29^. We were therefore interested in testing whether FLCN affects the endocytic trafficking of EGFR. Isogenic UOK257 FLCN-deficient and FLCN-replete cell lines were starved of amino acids and growth factors, stimulated with EGF ligand, and then fixed for IF at defined time points post stimulation in order to follow EGFR endocytic trafficking. Confocal microscopy of cells co-labelled with antibodies recognizing EGFR and a marker of either early endosomes, Early endosome antigen 1 (EEA1), or late endosomes/lysosomes, lysosomal-associated membrane protein 1 (LAMP1) revealed the localization of EGFR within the cell (Fig. 2b,d). The percentage of EGFR co-localizing with either EEA1 or LAMP1 at each time point was analysed and quantified with Image J software. We observed that EGFR internalization was fast in UOK257 cells, with ∼30% of EGFR co-localizing with EEA1-positive early endosomes 5 min after EGF stimulation in both UOK257 FLCN^−/−^ and FLCN-replete cell lines (Fig. 2a). These data suggest that FLCN does not affect EGFR internalization. However, 15 min after EGF stimulation, the percentage of EGFR in early endosomes (EEA1) decreased in FLCN WT cells but not in FLCN^−/−^ cells, indicating that the loss of FLCN slows trafficking through the EEA1-positive early endosomes. The delay in endocytic trafficking and accumulation of EGFR in early endosomes in FLCN^−/−^ cells was still significant 30 min after EGF stimulation. These results suggest that FLCN plays an important role in the movement of EGFR out of early endosomes.

Consistent with the finding that FLCN WT cells have faster trafficking of EGFR through the early endosomes, we observed in UOK257 FLCN WT cells that EGFR had already reached the LAMP1-positive late endosomes/lysosomes 30 min after EGF stimulation (Fig. 2c). At the 30 min time point, FLCN^−/−^ cells exhibited only 20% EGFR and LAMP1 co-localization compared to 30% in the FLCN WT cells. The decreased amount of EGFR in the late endosomes/lysosomes in the FLCN^−/−^ cells suggests that EGFR traffics more slowly from the early to late endosomes in FLCN^−/−^ cells compared to FLCN WT cells. After 60 min, the percentage of EGFR and LAMP1 co-localization was equal in both FLCN^−/−^ and FLCN WT cell lines (Fig. 2c). Taken together, these results indicate that FLCN WT expressing cells favour a fast endocytic trafficking of EGFR to the lysosomes for degradation, possibly decreasing EGFR expression and signalling. In contrast, FLCN^−/−^ cells have an accumulation of EGFR in the early endosomes (from where EGFR still signals) resulting in increased and prolonged EGFR signalling.

### Loss of FLCN results in increased EGFR signalling

**Figure 3:**
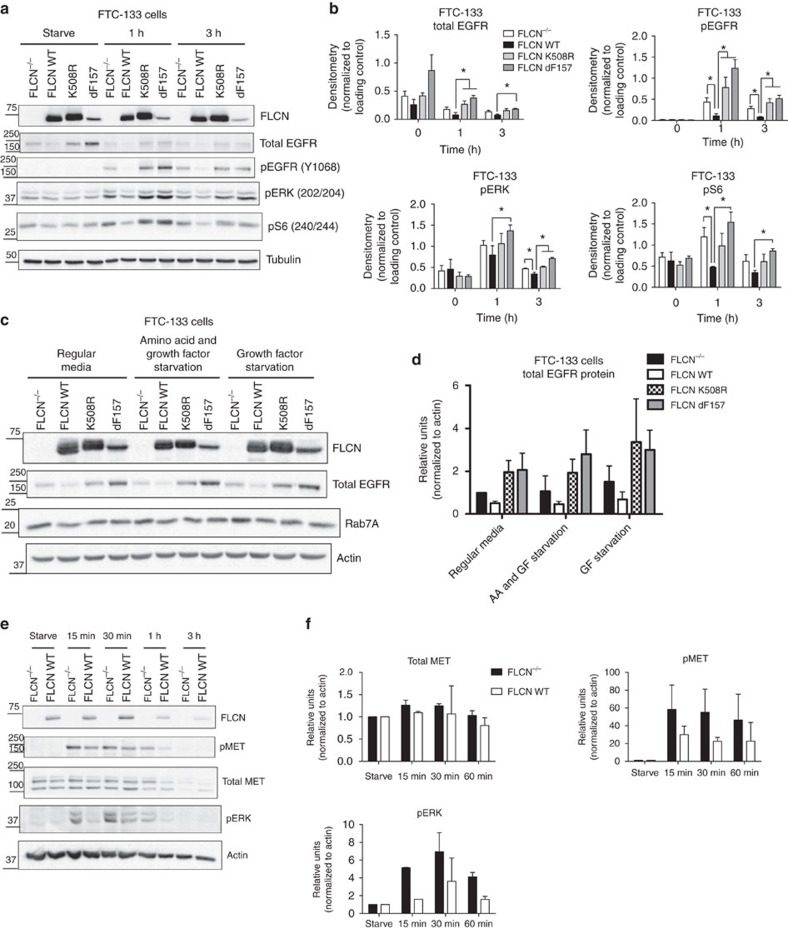
Ligand-dependent EGFR and MET signalling is increased in FLCN-deficient cells and cells expressing a tumour-associated FLCN mutant compared to FLCN-replete cells. (**a**) FLCN-deficient FTC-133 cells (FLCN^−/−^) and isogenic cells replete with wild type FLCN (FLCN WT) or the tumour-associated mutants FLCN K508R and FLCN d157 were starved for 2.5 h and stimulated with 20 ng ml^−1^ of EGF for the indicated time points (1 or 3 h). A representative western blot demonstrating decreased phospho-EGFR (pEGFR), phospho-ERK (pERK) and phospho-S6 (pS6) signalling in FTC-133 FLCN WT-replete cells compared to FLCN-deficient or tumour-associated mutant cells is shown. (**b**) The densitometry of the bands in panel A was determined with BioRad Image Lab Software, and normalized to the loading control (either actin or tubulin). The data are presented as the mean±s.e.m., and * indicates statistical significance, one-tailed *t*-test, *P*<0.05. (**c**) FLCN-replete cells (FLCN WT) express less total EGFR compared to cells that are FLCN-deficient (FLCN^−/−^) or express a tumour-associated mutant form of FLCN (FLCN K508R and d157). FTC-133 cells were plated at the same density and grown asynchronously in regular media, or starved for either (1) 2.5 h in growth factor and amino-acid-depleted RPMI media, or (2) overnight in DMEM serum-free (SF) media. The expression of total EGFR and Rab7A was determined by western blot of cell lysates. (**d**) Bar graph depicting the densitometry of the western blot bands normalized to the actin loading control (densitometry was evaluated using BioRad Image Lab Software). The data presented are the means±s.d. from three independent experiments. (**e**) FLCN-deficient FTC-133 cells (FLCN^−/−^) and isogenic cells replete with wild-type FLCN (FLCN WT) were starved as in **a** and stimulated with 25 ng ml^−1^ of HGF for the indicated time points. A representative western blot demonstrating increased phospho-MET (pMET), and phospho-ERK (pERK) signalling in FLCN-deficient cells is shown. (**f**) The densitometry of the bands in **e** was determined with BioRad Image Lab Software, normalized to the loading control (actin), and expressed relative to starved samples. The data are presented as the mean±s.e.m.

As shown in the previous experiments, FLCN interacts with and acts as a GAP for Rab7A. We hypothesized that FLCN promotes Rab7A function(s) in the cells. One of the functions of Rab7A in cells is to suppress ligand-dependent EGFR activation^28^,^29^,^34^. We were therefore interested in determining whether FLCN’s association with Rab7A affected EGFR signalling and expression. We used a second FLCN-deficient cancer cell line, FTC-133 cells, to examine EGFR signalling following amino acid and growth factor starvation and stimulation with EGF ligand. After stimulating with EGF ligand (20 ng ml^−1^) FLCN null cells (FTC-133 FLCN^−/−^) and cells expressing tumour-associated mutant forms of FLCN (FTC-133 K508R and FTC-133 dF157) have increased levels of phosphorylated EGFR (pEGFR) compared to cells expressing FLCN WT (Fig. 3a,b). The increase in phosphorylated EGFR in FLCN^−/−^ and tumour-associated mutants was most pronounced at 1 h after EGF treatment, but remained high at the 3 h time point (Fig. 3a,b). The expression of phosphorylated ERK (pERK) and S6 (pS6), which are downstream in the pEGFR signalling cascade, was also higher in the FLCN^−/−^ and tumour-associated mutant cells than in the cells expressing FLCN WT (Fig. 3a,b). These results suggest that loss of the FLCN tumour suppressor results in increased pEGFR signalling. A decrease in phospho-AKT was not observed in FTC-133 cells replete with FLCN WT, but this was expected because FTC-133 cells are PTEN null and do not regulate pAKT properly^35^.

FLCN^−/−^ cells have increased EGFR expression, relative to FLCN WT cells (Fig. 3c,d). This decrease in total EGFR expression was seen under normal cell growth conditions and under different starvation conditions (overnight growth in serum-free media and 2.5 h of starvation in RPMI 1640 media without growth factors and amino acids). The expression level of endogenous Rab7A was the same across all of the different growth conditions (regular media, serum-free media or media without growth factors and amino acids) and was not affected by expression of FLCN WT or the tumour-associated mutants (Fig. 3c). Although the focus of this study was to examine the role of the FLCN–Rab7A interaction on EGFR trafficking and signalling, it is possible that additional receptor tyrosine kinases (RTKs) are also affected. We starved and stimulated FTC-133 cells with hepatocyte growth factor (HGF) and examined pMET levels, since the MET receptor is mutated frequently in papillary renal tumours, one of the histological subtypes observed in the renal tumours of BHD patients^36^,^37^,^38^. Our data demonstrate that stimulation with HGF results in elevated pMET and pERK expression in FLCN^−/−^ cells compared to the isogenic FLCN WT cells (Fig. 3e,f).

### Rab7A decreases EGFR signalling in FLCN^−/−^ cells

**Figure 4:**
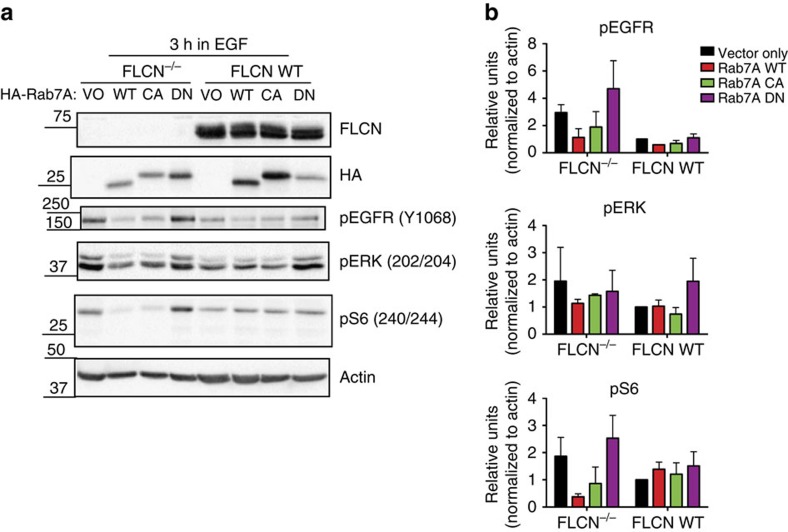
Expression of Rab7A phenocopies the effect of FLCN on ligand-dependent EGFR activation. (**a**) FLCN-deficient FTC-133 cells (FLCN^−/−^) and wild-type FLCN-replete cells (FLCN WT) were stably infected with viruses expressing a vector, wild-type Rab7A or the constitutively active (Q67L) or dominant-negative (T22N) Rab7A mutants (indicated as vector only (VO), WT, CA and DN). After 2.5 h of starvation, the cells were stimulated with 20 ng ml^−1^ EGF for 3 h and protein lysates were immunoblotted as indicated. A representative western blot demonstrating decreased pEGFR, pERK and pS6 signalling in FLCN^−/−^ cells expressing WT or CA Rab7A. (**b**) Quantification of western blot signal intensity from two independent experiments, presented as the as the mean±s.d. The densitometry of the bands was determined with BioRad Image Lab Software, normalized to the loading control (actin), and expressed relative to the FLCN replete cells (FLCN WT) infected with the vector only.

EGFR expression and signalling is elevated in cells lacking the WT FLCN tumour suppressor. We showed that FLCN interacts with Rab7A and it is known that Rab7A functions in EGFR recycling and degradation. In order to establish a causal relationship between the FLCN–Rab7A interaction and suppression of EGFR signalling by FLCN, we stably expressed Rab7A WT, or the CA or DN mutants in FTC-133 cells. Expression of either Rab7A WT or CA (compared to cells expressing the vector only (VO)) decreased pEGFR and downstream signalling molecules (pERK and pS6) in FLCN^−/−^ cells, but had little effect on pEGFR signalling in the FLCN WT cells (Fig. 4a,b). In addition, it appears that expression of a DN form of Rab7A may phenocopy the absence of FLCN, by increasing pERK signalling in FLCN-reconstituted cells (Fig. 4a). These data suggest that FLCN’s ability to regulate EGFR signalling is mediated through FLCN’s interaction with Rab7A.

### The FLCN and Rab7A functional interaction is conserved

To determine whether the FLCN and Rab7A functional interaction is conserved across species, we engineered *S. pombe* strains in which the FLCN (*bhd1*Δ) and the Rab7A (*ypt71*Δ and *ypt7*Δ) homologues were deleted completely. *S. pombe* has two close homologues of the human Rab7A protein, Ypt7 and Ypt71, which display 65% and 55% identity, and 80% and 75% similarity to Rab7A, respectively. We chose to study both Ypt7 and Ypt71 proteins to test if either of the two functionally interacts with Bhd1. All single mutant strains (*bhd1*Δ, *ypt71*Δ, and *ypt7*Δ) were constructed in an auxotrophic (*leu*^*−*^, *ade*^*−*^, *ura*^*−*^) background. Amino-acid deprivation and mating efficiencies were used as phenotypes for ascertaining functional overlap between Bhd1 and Ypt7 or Ypt71 proteins.

**Figure 5:**
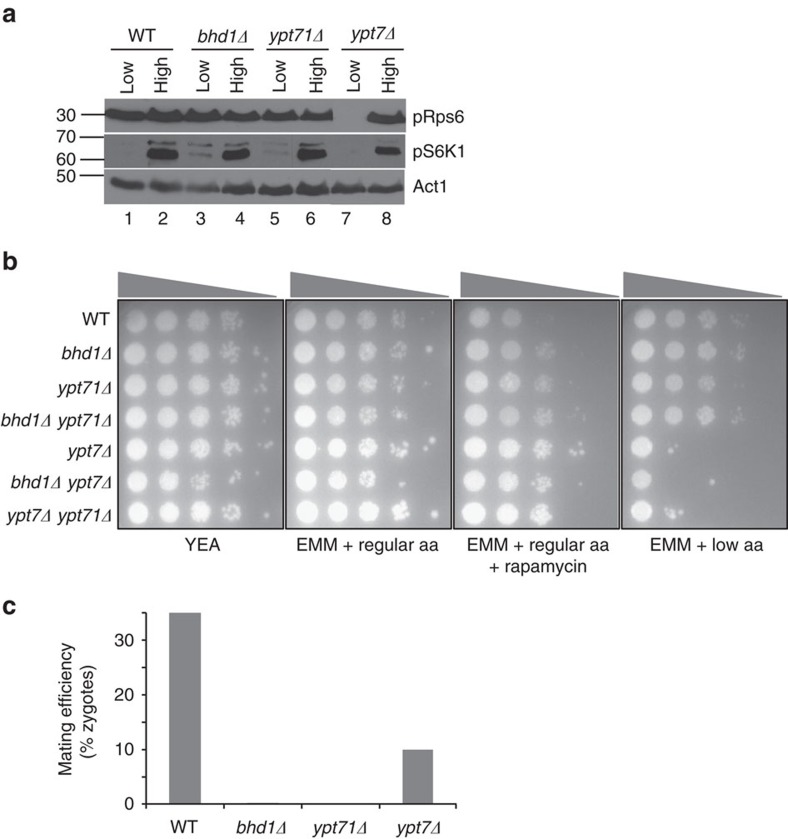
The *S. pombe *homologues of human FLCN *(bhd1)* and Rab7A (*ypt71*) function in the same genetic pathway. (**a**) Cells from the indicated genotypes were grown to log phase in minimal media (EMM) supplemented with amino acids at regular (225 mg l^−1^) concentration, and then transferred to either EMM supplemented with amino acids at low (45 mg l^−1^) or high (1,125 mg l^−1^) concentration. After 90 min, samples were taken and subjected to western blot analysis using antibodies against the phosphorylated form of Rps6 (pRps6) and S6K1 (pS6K1), which are direct substrates of TORC1 in the fission yeast and humans. Actin was used as a loading control. (**b**) Wild type (WT) and mutant strains were grown at 32 °C in liquid minimal media (EMM). Once in log phase, cells were spotted in a 10-fold concentration gradient on rich (YEA) or minimal (EMM) media supplemented with amino acids (aa) at regular (225 mg l^−1^) or low (45 mg l^−1^) concentration. Rapamycin (Rap, 200 ng ml^−1^) was added to EMM media supplemented with regular amino acids. The grey triangles represent the concentration gradient of cells spotted on each plate. Strain genotype is indicated on the left column. (**c**) For each indicated genotype, an equal number of cells of opposite mating types were crossed on EMM minus nitrogen plates. The mating efficiency was determined by calculating the percentage (%) of zygotes formed for each cross out of total number of cells. At least 400 cells were counted for each measurement.

All single mutant strains were viable and had no growth defect. We observed that loss of Bhd1 and Ypt71, but not Ypt7, resulted in increased TORC1 activity, as determined by an increase in Rps6 and p70 S6K phosphorylation levels when cells were deprived of amino acids (Fig. 5a, compare lanes 1, 3, 5 and 7). Compatible with this difference in TORC1 regulation by Bhd1/Ypt71 and Ypt7 are the strain differences in amino-acid requirements for growth. While all single mutant strains were viable and had no growth defects in the presence of regular amino-acid concentration (rich media (YEA) or minimal media (EMM) supplemented with regular concentration of amino acids), their response to low amino-acid concentration differed. Strains lacking Ypt7 (*ypt7*Δ, *bhd1*Δ *ypt7*Δ, *ypt71*Δ *ypt7*Δ) displayed a significant growth defect in the low amino-acid condition (EMM plates supplemented with low concentration of amino acids) compared to WT strains, or *bhd1*Δ and *ypt71*Δ strains (Fig. 5b). To determine whether these proteins are related to TORC1 signalling, we treated *bhd1*Δ, *ypt71*Δ, *ypt7*Δ and double deletion strains with 200 ng ml^−1^ of rapamycin and found that all of the strains grew better than WT cells (Fig. 5b). These data suggest that Bhd1 and Ypt71 (but not Ypt7) functionally interact and, in agreement with the mammalian cell data, negatively regulate TORC1 activity in response to amino-acid deprivation.

To corroborate the functional interaction of Bhd1 and Ypt71 with an orthogonal assay, we measured the mating efficiency of WT, *bhd1*Δ, *ypt71*Δ and *ypt7*Δ cells by determining the percentage of zygotes formed in each cross. In the fission yeast, mating proceeds by the secretion of mating pheromone from one cell and its binding to a G-coupled cell surface receptor on a cell of the opposite mating type. Upon pheromone binding, several physiological changes occur that are essential for mating, including suppression of TORC1 activity^39^. Unlike *ypt7*Δ, mating of cells from strains lacking Ypt71 or Bhd1 produced no zygotes (Fig. 5c). Similar to the amino-acid deprivation data, these results show that Ypt71 and Bhd1 functionally interact during mating, perhaps by negatively regulating TORC1 activity. Our data suggest that Ypt71 (and not Ypt7) is the functional homologue of the mammalian Rab7A and that the functional interaction between Rab7A and FLCN is conserved across species.

### EGFR is activated in FLCN^−/−^ mouse and human tumours

**Figure 6:**
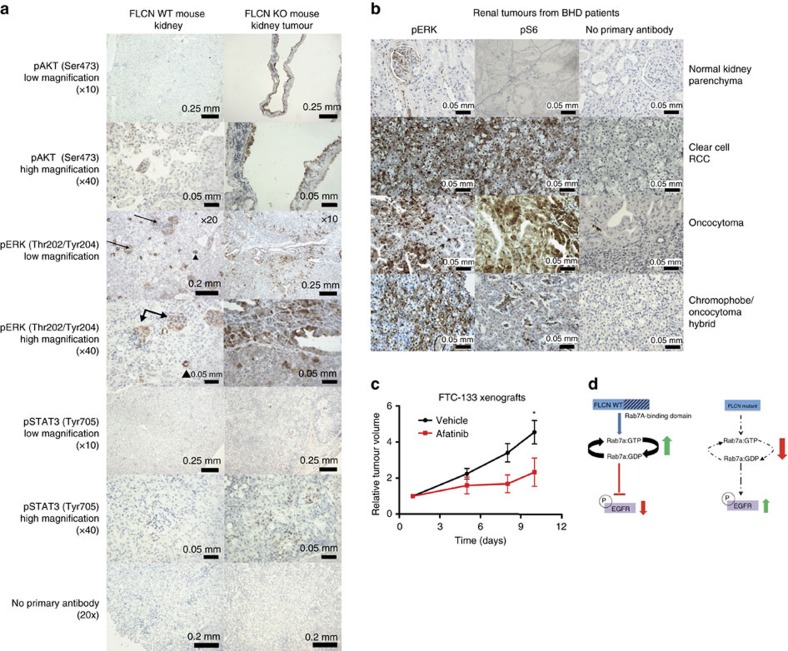
EGFR signalling is elevated in FLCN^−/−^ mouse tumours and treatment with an EGFR inhibitor suppressed tumour growth. (**a**) Immunohistochemical staining of littermate mouse FLCN WT kidney (Flcn ^flox/flox^) and mouse kidney tumours generated in the FLCN kidney KO (Sglt2-Cre;Flcn^flox/flox^) mouse model. The cysts and tumours in FLCN KO kidney tumours strongly express pAKT, pERK and pSTAT3 (best seen in the high magnification (× 40) images). The glomeruli (arrows) and collecting ducts (arrowheads) of normal (FLCN WT) mouse kidneys express pERK, but pAKT and pSTAT3 staining was almost absent, except for a few positive cells. Scale bars, 0.25 mm at × 10 magnification; 0.2 mm at × 20 magnification; 0.05 mm at × 40 magnification. (**b**) Immunohistochemical staining of a panel of human kidney tumours representing the clear cell, oncocytoma and chromophobe/oncocytoma histologies demonstrated increased pERK and pS6 signalling compared to normal kidney. The glomeruli and collecting ducts express pERK, but otherwise pERK and pS6 staining was almost absent in normal kidney tissue. Scale bars, 0.05 mm. (**c**) FLCN^−/−^ FTC-133 cells were injected subcutaneously into nude mice and once tumours were established, the mice were treated daily with vehicle or Afatinib (20 mg kg^−1^ for 6 days followed by 15 mg kg^−1^ for 3 days). The tumour volume relative to the size of the tumour on day 1 of treatment is presented and the error bars are the mean±s.e.m. (*n*=9 mice in the vehicle group, and *n*=8 mice in the Afatinib group). The relative tumour volume at the end of the study (day 10) is significantly different between the vehicle and Afatinib treated mice, * indicates significance, *t*-test, *P*<0.01. (**d**) Active Rab7A accelerates the endocytic trafficking of internalized EGFR to the lysosome for degradation, resulting in reduced EGFR signalling (phosphorylated EGFR, ERK and S6). When cells lose the tumour suppressor function of FLCN due to a germline mutation in the FLCN gene (BHD disease), Rab7A GTP-to-GDP turnover is decreased. A decrease in Rab7A activity slows the endocytic trafficking of EGFR, resulting in prolonged and elevated phosphorylated EGFR and downstream signalling.

In order to determine whether the increase in EGFR signalling observed in FLCN^−/−^ cell lines is also present *in vivo*, we examined pAKT, pERK and pSTAT3 expression in a genetically engineered mouse model of BHD kidney cancer (*Flcn*^flox/flox^/*Sglt2-Cre* mouse model^40^) and in the renal tumours of patients with BHD disease. The *Flcn*^flox/flox^/*Sglt2-Cre* mouse model utilizes the Cre loxP system to knock out FLCN specifically in the proximal tubules of the kidney. All of the FLCN KO (*Flcn*^flox/flox^/*Sglt2-Cre*) mice over 6 months of age develop cystic kidneys and more than 50% of the mice also develop renal tumours^40^. Normal FLCN WT mouse kidneys (*Flcn*^flox/flox^) express very low levels of pAKT and pSTAT3 (Fig. 6a). pERK was highly expressed in the collecting ducts and glomeruli of normal (FLCN WT) mouse kidneys, but was not expressed in the kidney tubules (Fig. 6a). In contrast, pAKT was expressed exclusively in the renal cysts in FLCN KO mouse kidneys, while pSTAT3 and pERK were highly expressed throughout the cysts and in the renal carcinomas (Fig. 6a). Similarly, IHC analysis of human renal tumours obtained from BHD patients demonstrated that pERK and pS6 are highly expressed in several histological subtypes of renal cell carcinomas, including clear cell, oncocytoma, chromophobe and mixed oncocytoma/chromophobe (Fig. 6b and Table 1). These data support our *in vitro* data and suggest that elevated EGFR signalling may contribute to kidney tumorigenesis following FLCN loss. To determine whether suppression of EGFR signalling is sufficient to inhibit the growth of FLCN^−/−^ tumours, FTC-133 cells were injected subcutaneously into nude mice (5 × 10^6^ cells per mouse). The FTC-133 (FLCN^−/−^) cells were chosen because they reproducibly produce xenograft tumours with a short latency (3–5 weeks). Once tumours were established, the mice were treated daily with Vehicle or Afatinib. Afatinib significantly slowed the growth of FTC-133 (FLCN^−/−^) xenograft tumours compared to the Vehicle-treated tumours (Fig. 6c), suggesting that the increased pEGFR signalling observed *in vitro* is also important for *in vivo* growth of FLCN^−/−^ tumours.

## Discussion

To gain insight into the biochemical functions of FLCN, we purified protein complexes and identified Rab7A, a small GTPase important for endocytic trafficking and lysosomal degradation of EGFR, as a novel FLCN interacting protein. Furthermore, we provided biochemical evidence indicating that FLCN WT protein, but not a tumour-associated missense FLCN mutant, increased the GTP hydrolytic activity of Rab7A. Consistent with Rab7A’s function in endosomal trafficking of EGFR, we demonstrated that FLCN^−/−^ cells have delayed trafficking of EGFR from the early endosomes to the late endosomes/lysosomes and that FLCN^−/−^ cells display increased and prolonged EGFR activation compared to FLCN-replete cells, in a Rab7A-dependent manner. This is not an *in vitro*-only phenomenon; renal cell carcinomas growing in FLCN KO mouse kidneys display strong activation of the EGFR signalling pathway and treatment of FTC-133 FLCN^−/−^ mouse xenografts with the EGFR inhibitor Afatinib slowed tumour growth. Finally, the genetic interaction between *S. Pombe* FLCN and Rab7A orthologs corroborated the functional interaction discovered in mammals and indicated that the pathway is evolutionary conserved. Our model (Fig. 6d) hypothesizes that FLCN^−/−^ cells have decreased Rab7A GTP-to-GDP turnover and decreased endosomal trafficking of EGFR. The increase in pEGFR signalling in FLCN^−/−^ cells is at least partly due to EGFR’s ability to stimulate downstream signalling cascades from within endosomes^31^.

Our work indicates that regulation of EGFR signalling by FLCN is, at least in part, Rab7A-dependent. This is compatible with the notion that FLCN acts as a GAP for Rab7A, the latter being an important regulator of endocytic trafficking^28^,^29^,^30^. It is likely that regulation of Rab7A by FLCN contributes to several cellular processes other than EGFR signalling. A recent study demonstrated that knocking down FLCN results in reduced maturation of autophagosomes and reduced autophagic flux^11^. Although not addressed in the above study, it is possible that this is a Rab7A-dependent process, since Rab7A is important for the fusion of lysosomes with autophagosomes^41^.

Although we focused on the functional interaction between FLCN and Rab7A, we provided evidence that FLCN forms putative complexes with additional Rab proteins (Rab7B, Rab35 and Rab9A), albeit with a lower affinity than with Rab7A. Many of these additional Rabs function in the regulation of the endocytic, recycling and secretory pathways^23^,^42^,^43^. The difference in binding affinity between the Rab GTPases could be due to differences in the protein complex stoichiometry or due to specific growth conditions that favour binding to one Rab GTPase over another. For example, FLCN was shown to bind the Rag GTPases in response to amino-acid stimulation following starvation^17^,^18^. It is also possible that the other FLCN-interacting Rab GTPases that we identified here (Rab9A and Rab35), in addition to Rab7A, contribute to the regulation of EGFR by FLCN.

To test whether the functional interaction between FLCN and Rab7A is evolutionary conserved, we took advantage of the fission yeast system. *S. pombe* has one FLCN homologue (Bhd1), and two Rab7A homologues (Ypt7 and Ypt71), which are similar in amino acid sequence and function to their mammalian homologues^44^,^45^,^46^,^47^. Bhd1, like FLCN, regulates TORC1 activity, and Ypt7 and Ypt71, similar to Rab7A, are important for vacuolar biogenesis and late vesicle fusion to vacuoles, the functional equivalents of the mammalian late endosomes and lysosomes, respectively^45^,^47^,^48^. Previous work demonstrated that both Ypt7 and Ypt71 are homologues of Rab7A, and even though both localize to vacuolar membranes, their absence and overexpression resulted in antagonistic vacuolar phenotypes^45^. We found a genetic interaction between Bhd1 and Ypt71 that supports a role for these proteins in the regulation of Torc1 signalling. Our observations suggest that Bhd1 and Ypt71 negatively regulate the Torc1 pathway under low amino acid growth conditions. These findings are in agreement with the function of FLCN as a tumour suppressor gene and are consistent with the functional interaction between FLCN and Rab7A in mammalian cells.

FLCN has been shown to bind FNIP1, FNIP2, the Rag GTPases A and C/D, GABARAP and plakophilin-4 (refs 11, 12, 13, 14, 15, 16, 17, 18), but the biological significance of each of these interactions with regards to the tumour suppressor function of FLCN is under investigation. In contrast to our results indicating that the absence of FLCN enhances TORC1 activity, Tsun *et al*. and Petit *et al*.^17^,^18^ demonstrated that FLCN was required for the activation of mTORC1 at the lysosome by amino acids (due to FLCN’s GAP or GEF activity for the Rag GTPases). This apparent discrepancy raises the hypothesis that different stimuli or growth conditions (for example, amino-acid stimulation versus growth factor stimulation) regulate FLCN’s ability to bind different GTPases or control FLCN’s recruitment to specific cellular locations, and may exert opposing effects with regards to mTORC1 activity. However, if FLCN activates mTORC1 signalling at the lysosome, then loss-of-FLCN function would lead to suppression of mTORC1, which seems to contradict FLCN’s role as a tumour suppressor protein. Our results demonstrating that FLCN decreases mTORC1 signalling (a decrease in pS6) in response to growth factors (that is, EGF ligand) are compatible with FLCN’s role as a tumour suppressor protein and suggest that the main mechanism leading to tumorigenesis in FLCN-deficient human cells may be linked, at least in part, to enhanced receptor tyrosine kinase signalling which increases TORC1 activity.

Our results suggest that the tumour suppressor function of FLCN is, at least in part, due to its ability to inhibit the oncogenic signalling of EGFR, by acting as a GAP protein for Rab7A and therefore modulating the fate of receptor trafficking following endocytosis. Although not examined in our current work, it is likely that FLCN-dependent regulation of the endocytic pathway is important for the expression and/or function of additional cell surface trans-membrane RTKs and non-RTK receptors. For example, Rab7A has been shown to affect the expression, trafficking, or signalling of several receptors in addition to EGFR, including VEGFR2, TrkA, HER2, MET and NRP-1 (refs 49, 50, 51, 52, 53). We provide evidence that FLCN regulates ligand-dependent activation of the MET receptor.

It is possible that post-translational modifications further fine-tune the interactions between FLCN-Rab7A-EGFR. For example, it has been shown that PTEN modulates EGFR late endocytic trafficking and degradation by dephosphorylating Rab7A (ref. 54). In our current work, we tested the effect of FLCN phosphorylation in residues S63 and S73 and found that phosphorylation on these sites did not alter FLCN’s interaction with Rab7A. It is nevertheless formally possible that FLCN phosphorylation in residues other than S62/S73 or Rab7A post-translational modifications do regulate the interaction between FLCN-Rab7A.

The translational significance of our work is direct. Our *in vivo* study demonstrated that inhibiting EGFR signalling with afatinib was sufficient to slow the growth of FLCN^−/−^ tumours, but did not result in tumour regression. Targeting several cell surface RTKs simultaneously, in addition to EGFR, or inactivating intracellular kinases that function as converging hubs of deregulated signalling pathways may be an effective therapeutic strategy for treating FLCN-dependent renal cell cancers.

## Methods

### Cell lines and cell culture

The UOK257 renal carcinoma cell line (a generous gift from Drs Marston Linehan and Laura Schmidt, NCI/NIH) is a non-commercial cell line originally derived from the clear cell renal tumour of a BHD patient^55^,^56^. The FLCN-deficient human follicular thyroid carcinoma cell line FTC-133 was originally obtained from ATCC and was generously provided by Dr Cyril Benes. The UOK257 and U20S cells were grown in Dulbecco’s Modified Eagle Medium (DMEM) and the FTC-133 cells in DMEM/Nutrient mixture F-12 (F12), both supplemented with 10% fetal bovine serum, penicillin, streptomycin and L-glutamine (Invitrogen, Carlsbad, CA). Mycoplasma testing was performed to ensure that the cells were mycoplasma negative. The UOK257 and FTC-133 cells were infected with retroviruses encoding for the pBABE-puro vector, FLCN WT, Flag-FLCN WT or the FLCN tumour-associated mutants^33^. FTC-133 cells were also infected with retroviruses encoding the pBABE-hygro vector, HA-Rab7A, HA-Rab7A T22N or HA-Rab7A Q67L. UOK257 cells were selected in 2 μg ml^−1^ of puromycin and FTC-133 cells were selected in 3 μg ml^−1^ of puromycin and 0.25 mg ml^−1^ hygromycin. The presence of protein tags is as indicated in the figures and figure legends. Non-tagged protein expression (that is, FLCN) is implied when protein expression is indicated without reference to any tag. Supplementary Figure 4 demonstrates the level of expression of exogenous FLCN WT after infection compared to endogenous expression in a panel of cell lines.

### Plasmids

The plasmids and oligonucleotides used to generate FLCN WT, FLCN K508R and FLCN dF157 retroviruses were previously described^33^. Flag-FLCN WT was PCRed using FLCN WT DNA and oligonucleotides (5′-GCGC GAATTCA GTT CCG AGA CTC CGA GGC TGTG-3′ and 5′-GCGC GGATCC GCCACC ATG GAT TAC AAA GAT GAT GAT GAT AAA AAT GCC ATC GTG GCT CTC TG-3′) and ligated into the pBABE-puromycin vector plasmid with BamHI and EcoRI restriction sites. FLCN WT, FLAG-FLCN WT and the FLCN K508R mutant were cloned into the pCDNA3.1 backbone with BamHI and EcoRI. The HA-eGFP-Rab7A WT and the HA-eGFP-Rab7A Q67L mutant were cloned into the pCDNA3 backbone (previously described^57^) and obtained from Addgene (plasmid 28047 and plasmid 28049). The HA-eGFP-Rab7A T22N mutant and Rab9A-HA-GFP were cloned into pEGFP-C1 (Addgene plasmids 12660 and 12663 (ref. 58)). Flag-Rab8A was cloned into pcDNA3.1neo (Addgene plasmid 46783 (ref. 59)). Rab7B-Myc-DDK (RC202283) and Rab35-Myc-DDK (RC201932) in pCMV6-Entry were purchased from OriGene Technologies, Inc. (Rockville, MD). HA-Rab7A in pcDNA3 was produced by PCR using HA-eGFP-Rab7A WT as template and with 5′-GCGCGGATCCATGACCTCTAGGAAG-3′ and 5′-GCGCGAATTCAGCAACTGCAGCTTTCTG-3′ oligonucleotides. HA-Rab7A T22N was created using HA-eGFP-Rab7A T22N DNA as template and HA-Rab7A Q67L was created using HA-eGFP-Rab7A Q67L DNA as template and both PCR products used 5′-GCGCGGATCCGCCACCATGTACCCATAC-3′ and 5′-GCGCGAATTCAGCAACTGCAGCTTTCTG-3′ as oligonucleotides. The HA-Rab7A, HA-Rab7A T22N and HA-Rab7A Q67L PCR products were then restricted and ligated into the pBABE-hygromycin vector plasmid.

### Cell fractionation and protein purification

Approximately 100 million cells (UOK257 vector only and UOK257 cells replete with Flag-FLCN WT) were fractionated into nuclear, cytoplasmic and membrane fractions. Briefly, cells were washed in PBS and collected via scraping. The cells were then pelleted (1,200 r.p.m. for 4 min) and washed twice in RBS buffer (10 mM HEPES, 10 mM NaCl, 1.5 mM MgCl_2_) containing protease and phosphatase inhibitors. The cell pellet was resuspended in RBS buffer on ice for 10 min and then lysed with a Dounce homogenizer. When ∼95% of the cells were disrupted, the nuclei were pelleted by centrifuging at 380*g* for 10 min. The nuclei (pellet) was washed with RBS, pelleted, and extracted in EBC buffer (50 mM Tris pH8, 120 mM NaCl, 1% Nonidet P-40) containing protease and phosphatase inhibitors. The supernatant was then centrifuged at 150,000*g* for 1.5 h to pellet the membrane fraction. All fractions were washed and spun twice to remove any possible cross contamination between fractions. The membrane pellet was extracted in EBC buffer plus protease and phosphatase inhibitors. Flag-FLCN protein was IP’ed overnight at 4 **°**C from each of the fractions using anti-flag M2-agarose beads (Sigma-Aldrich, St Louis, MO). The beads were washed with NET-N buffer (100 mM NaCl, 20 mM Tris-HCl pH8, 1 mM EDTA, 0.5% NP-40) and the protein complexes eluted in 80 mM glycine pH 2.5+2.5% SDS.

### Mass spectrometry

Proteins, affinity enriched with FLCN, were identified and quantified using a spectral counting approach essentially as described previously^60^. In brief, reduction and thiol alkylation was followed by purifying the proteins using MeOH/CHCl_3_ precipitation. Protein digest was performed with Lys-C and trypsin, and the peptides were subjected to microcapillary liquid chromatography tandem mass spectrometry (LC-MS2) on an Orbitrap Fusion mass spectrometer. MS2 spectra were assigned using a SEQUEST^61^ proteomics analysis platform. Based on the target-decoy database search strategy^62^ and employing linear discriminant analysis and posterior error histogram sorting, peptide and protein assignments were filtered to false discovery rate (FDR) of <1% (ref. 63).

### Transfections and IPs

U20S and 293T cells were transiently transfected with DNA using Polyjet (SignaGen Laboratories, Rockville, MD) according to the manufacturer’s instructions. Approximately 24–40 h after transfection, cells were lysed in EBC buffer containing protease and phosphatase inhibitors. Protein was immunoprecipitated from whole-cell extracts using 2 μg of FLCN antibody (#3697, Cell Signaling Technology, Danvers, MA) coupled to 10 μl of Protein A Dynabeads according to the manufacturer’s directions (Invitrogen Carlsbad, CA). The beads were washed in NET-N buffer and the protein complexes eluted in 20 μl of 0.1 M glycine pH 2.5 for 10 min at 70 °C. The FLAG-tagged FLCN phospho-mutants and the FLCN truncation mutants were immunoprecipitated using 10 μl of Anti-FLAG M2 magnetic beads (M8823, Sigma, St Louis, MO) according to the manufacturer’s instructions.

### Western blots and antibodies

Protein expression was detected by western blotting, as previously described^64^. Briefly, for cell lysis, RIPA buffer containing protease and phosphatase inhibitors was used. Proteins were separated by SDS–polyacrylamide gel electrophoresis electrophoresis, transferred to a PVDF membrane and detected with the cognate antibody. The following antibodies were used: anti-Pan Actin (1:10,000; Neomarkers, Fremont, CA); anti-total EGFR (1:1,000; sc-03, Santa Cruz, Dallas, TX); anti-pEGFR (1:1,000; ab5644, Abcam, Cambridge, MA); anti-HA tag (12CA5) (11 583 816 001, Roche, Germany). The anti-Rab7A (1:6,000; #R8779), anti-FLAG tag (1:10,000; #F1804) and anti-Tubulin (1:5,000; #T9026) antibodies were from Sigma-Aldrich (St Louis, MO). The anti-FLCN antibody (1:3,000; #3697), anti-pERK (1:2,000; #4370 and 1:2,000; #9101), anti-pS6 (1:2,000; #5364), anti-HA (1:2,000; #3724), anti-pMET (1:1,000; #3129), total MET (1:1,000; #3148) and IgG control (#3900) antibodies were from Cell Signaling Technology (Danvers, MA). Western blots were developed using the Bio-Rad ChemiDoc system and densitometry was analysed with BioRad Image Lab Software (Bio-Rad Laboratories, Hercules, CA). Uncropped scans of the most important blots are contained in Supplementary Fig. 6.

### IHC in FLCN^−/−^ mouse model and human patient samples

Mouse kidney tumours were obtained from the previously described C57BL/6 *Flcn*^flox/flox^/*Sglt2-Cre* mouse model of BHD kidney cancer (*n*=4 mice, male and female) and normal kidneys (*n*=4 mice) were obtained from FLCN WT mice^40^. All animal experiments were performed according to the standards of IACUC-approved protocols and the approval of MGH Subcommittee of Research and Animal Care (SRAC). RCC samples and matching normal kidney tissue as control were obtained from patients with BHD disease. All patients provided informed consent for tumour collection and analysis per IRB-approved protocol. Mouse and human tissues were deparaffinized, rehydrated and unmasked in sodium citrate buffer (incubated at 95 °C for 20 min). Endogenous peroxidase activity was blocked with 3% hydrogen peroxide, and non-specific binding of the primary antibodies was blocked with 2% goat serum and 1% BSA in TBST. The primary antibodies pAKT (1:100; #4060), pERK1/2 (1:100; #4370), pS6 (1:2,000; #5364) or pSTAT3 (1:100; #9145) (Cell Signaling Technology, Danvers, MA) were incubated with the tissues at 4 °C overnight. The primary antibodies were washed with TBST and the secondary antibody (Biotinylated anti-Rabbit) was applied to the tissues for 1 h at room temperature. The secondary antibody was washed and the tissues were developed with the ABC Kit and DAB Kit (Vector Laboratories, according to the manufacturer’s instructions), counterstained with haematoxylin, dehydrated and mounted.

### Rab7A GTPase activation assay

The total amount of inorganic phosphate produced by Rab7A’s hydrolysis of GTP was measured using a commercially available kit and following the manufacturer’s instructions (Innova Biosciences, #602-0120, Cambridge, UK). To purify full-length FLCN, FLCN K508R mutant or Rab7A proteins, we transfected 293T cells with DNA plasmids expressing the corresponding proteins, lysed with RIPA lysis buffer containing phosphatase and protease inhibitors, and immunoprecipitated the transfected proteins from lysates. To purify FLAG-tagged WT or mutant FLCN, we used anti-FLAG M2 affinity gel (Sigma-Aldrich, St Louis, MO). Untagged FLCN WT and FLCN K508R proteins were purified by IP using anti-FLCN antibody (Cell Signaling Technology #3697) bound to protein A sepharose CL-45 beads (GE Healthcare). To purify Rab7A, we used anti-HA antibodies 12CA5 (#11583816001, Roche, Germany) or HA-Tag (C29F4) (#3724, Cell Signaling Technology, Danvers, MA) bound to protein A sepharose CL-45 beads. The beads (containing the purified protein) were washed five times with assay buffer and combined in the wells of a 96-well plate as indicated in each lane of Fig. 1d,e (10 μl of FLCN protein bound to beads and 30 μl of Rab7A protein bound to beads). All wells of the assay contained assay buffer and 0.5 mM GTP, except the wells containing the non-hydrolysable GTPγS, as indicated in Fig. 1d. The plates were incubated at 37 °C until completion of the reaction. For the GST protein purifications (used in Fig. 1e), full-length WT FLCN (GST-FLCN), a tumour-associated truncated mutant form of FLCN (GST-FLCN C9), or the GST vector alone (pGEX4T3) were purified from BL2 bacteria grown for 3 h at 30 °C after induction with 0.5 mM IPTG (Isopropyl β-D-1-thiogalactopyranoside, 367-93-1, Sigma, St Louis, MO) using Glutathione Sepharose 4B beads (#17075601, GE Helthcare Life Sciences, Pittsburgh, PA). Beads were combined together in reaction buffer and 0.5 mM GTP as described above. For each experiment, a fresh purification of all of the proteins was produced and equal amounts of beads were loaded into each well. The amount of free Pi was measured using the PiColorLock Gold reagent and read at a wavelength of 635 nm. Supplementary Fig. 5 demonstrates that in the presence of the phosphatase inhibitor NaF, FLCN WT increases the GTPase activity of Rab7A.

### Starvation and growth factor treatment of cells

FTC-133 cells were plated at similar densities and starved in either serum-free (SF) DMEM media overnight (Invitrogen, Carlsbad, CA) or in growth factor and amino-acid-depleted RPMI 1640 media for 2.5 h (US Biological Life Sciences, Salem, MA). The cells were stimulated after starvation with 20 ng ml^−1^ of EGF ligand (AF-100-15, PeproTech, Rocky Hill, NJ), or 25 ng ml^−1^ of HGF ligand (100-39, PeproTech, Rocky Hill, NJ) in growth factor and amino-acid-depleted RPMI 1640 media and collected at various time points (15 min, 30 min, 1 h and 3 h) for protein extraction and western blot.

### IF and confocal microscopy

For co-localization analysis of FLCN WT and Rab7A, U2OS cells were transfected with a vector only, or FLCN WT and HA-GFP-Rab7A WT in combination. The cells were fixed with 100% methanol for 10 min at −20 °C, and stained with an anti-FLCN antibody (1:1,000; #3697, Cell Signaling Technology (Danvers, MA)). For the EGFR trafficking experiments, UOK257 vector only and FLCN WT cells were starved for 2 h without growth factors and amino acids. Cells were stimulated with EGF ligand (1 μg ml^−1^) and fixed at various time points for IF. For the LAMP1 experiment, chloroquine diphosphate salt (100 μM, Sigma-Aldrich, St Louis, MO) was added during starvation and during EGF stimulation. The cells were washed, fixed as described above and incubated with anti-LAMP1 (1:200; #9091, Cell Signaling Technology, Danvers, MA) and anti-EGFR (1:500; #05-1047, Millipore, Temecula, CA) antibodies in IF diluent (0.5% Triton-X and 3% BSA in PBS). For the EEA1 experiment, cells were washed with PBS, fixed with 4% formaldehyde (methanol-free, Thermo-Scientific, Waltham, MA) for 15 min at room temperature, and then incubated with anti-EEA1 (1:500; #3288, Cell Signaling Technology, Danvers, MA) and anti-EGFR antibodies in IF diluent. The cells were washed and then incubated with anti-Mouse Cy3-conjugated (1:250; #715-165-150, Jackson ImmunoResearch Laboratories, West Grove, PA) and anti-Rabbit Alexa Fluor 488 (1:250; #A-11008, Invitrogen, Carlsbad, CA) secondary antibodies. The coverslips were mounted with Vectashield HardSet Mouting Medium with DAPI (Vector Laboratories, Burlingame, CA), and the cells visualized with a × 60 oil immersion lens on an inverted confocal microscope (Zeiss 710, Carl Zeiss Microscopy, Thornwood, NY).

### Xenograft tumours and afatinib treatment

Animal studies were conducted according to the guidelines of the MGH Institutional Animal Care and Use Committee. FLCN^−/−^ FTC-133 cells (5 × 10^6^) were injected sub-cutaneously into nude mice. Once tumours were established, the mice were treated daily by oral gavage with either the vehicle (0.5% methyl cellulose+0.4% Tween 80 (w/v)) or afatinib. Afatinib was given at 20 mg kg^−1^ for 5 days and then at 15 mg kg^−1^ for 3 days. The tumour volume was calculated using the following equation: (length (mm) × width^2^ (mm^2^))/2. The investigators involved in tumour size measurement were not blinded as to the treatment randomization of the mice. The number of animals used was calculated to exceed the number of animals needed to achieve statistical significance of *P*<0.05 with an 80% probability, estimating a 30% difference in means.

### Fission yeast strains and media

All *S. pombe* strains used in this study are listed in Supplementary Information (Supplementary Table 1). Standard cell growth, transformation and strain construction methods were used^65^,^66^,^67^.

### Fission yeast growth assay

*S. pombe* strains were grown in liquid Edinburgh minimal medium (EMM) media supplemented with 225 mg l^−1^ of amino acids (uracil, arginine, histidine, adenine, leucine) until log phase (OD_600_ of 0.8–1.2). An equal number of cells were spotted in a 10-fold concentration gradient onto rich media (YEA), EMM with normal (225 mg l^−1^) amino acids plus or minus 200 ng ml^−1^ Rapamycin (Sigma-Aldrich) or low (45 mg l^−1^) amino acids. Plates were incubated for 3–7 days at 32 °C.

### Measurement of fission yeast mating efficiency

Haploid cells of opposite mating types (with the same genetic background) were crossed on nitrogen-free minimal medium plates (EMM-N) and incubated for 48 h at 25 °C. The numbers of cells and zygotes were counted under a microscope. The mating efficiencies were determined by calculating the % of zygotes formed for each cross, out of the total number of cells. All data were calculated by counting at least 400 cells.

### Fission yeast protein extraction and western blot

Cells were grown to log phase (OD_600_ of 0.8–1.2) in EMM supplemented with 225 mg l^−1^ of uracil, arginine, histidine, adenine, leucine and then washed and transferred to EMM plus either low (45 mg l^−1^) amino acids or high (1125, mg l^−1^) amino acids. After 90 min of incubation, samples were taken and subjected to trichloroacetic acid (TCA) protein extraction. *S. pombe* cultures (5 ml) at an OD_600_ of 0.8–1.2 were pelleted just after the addition of 100% TCA and washed in 20% TCA. The pellets were lysed with three 45-s pulses in a MAgNA lyser following the addition of glass beads and 100 μl 12.5% TCA. Cell lysates were pelleted for 20 min at 13,200 r.p.m., washed in acetone, and dried at 37 °C for 15 min. Pellets were resuspended in 50 μl of a solution containing 1% SDS, 100 mM Tris-HCl (pH 8.0), and 1 mM EDTA. For western blotting, proteins were separated on a 12% SDS–polyacrylamide gel electrophoresis gel, transferred onto a nitrocellulose filter (Amersham), and probed with anti Phospho-S6 Ribosomal Protein (1:500; Ser235/Ser236; #2211) and Phospho p70 S6 Kinase (1:500; Thr389; #9205, Cell Signaling Technology, Danvers, MA) primary antibodies. Actin was used as a loading control (MS1295P Thermo).

### Data analysis and statistics

For the EGFR trafficking IF and confocal microscopy, pictures were processed using ImageJ 1.47v software and an additional co-localization plugin (Pierre Bourdoncle, Institut Jacques Monod, Service Imagerie, Paris) as previously described^30^. The background for each channel was determined by using the image from the no primary antibody control slide and then subtracted. The co-localization plugin generated a binary image of co-localized pixels whose intensities were higher than the background. The ‘min’ operation between the binary image of co-localization and the image of total EGFR fluorescence allowed the co-localized pixels to be converted to the real value of the EGFR fluorescence as a 32-bits image. A ratio of the fluorescence intensities for each image (co-localized EGFR versus total EGFR) was calculated for each field of view. For each time point, two randomly chosen fields of view (5–10 cells/area) were imaged with three Z-stack images in different planes (6 measurements). Three independent experiments were performed and all of the measurements were combined for analysis (18 measurements). Statistical differences between groups (Vector only and FLCN WT cells) were concluded by Student *t*-tests (or non-parametric Wilcoxon rank-sum tests when distributions were not normal). The Rab7A GTPase activity data were compared using ANOVA and Tukey’s multiple comparison post-tests. The relative tumour volumes in the FTC-133 xenograft study (comparing vehicle to Afatinib treatment) were compared with a two-way ANOVA with Bonferroni post-tests. All of the western blot densitometry data, GTPase activity data and the *in vivo* tumour growth assay were plotted and analysed using GraphPad Prism Software (Graph-Pad Software, San Diego, CA). Statistical significance was inferred at *P*<0.05.

### Data availability

All data included in this publication are available from the authors.

## Additional information

**How to cite this article:** Laviolette, L. A. *et al*. Negative regulation of EGFR signalling by the human folliculin tumour suppressor protein. *Nat. Commun.* **8,** 15866 doi: 10.1038/ncomms15866 (2017).

**Publisher’s note:** Springer Nature remains neutral with regard to jurisdictional claims in published maps and institutional affiliations.

## Supplementary Material

Supplementary Information

Supplementary Data 1

## Figures and Tables

**Table 1:** Immunostaining of human RCC tumours from BHD patients

**Tissue**	**pERK**	**pS6**
Normal kidney parenchyma	Glomeruli and collecting ducts are positive	
Patient 1—RCC chromophobe	++	+/−
*Patient 2—RCC clear cell	++	++
*Patient 2—RCC oncocytoma	++	++
†Patient 3—RCC oncocytoma	—	—
†Patient 3—RCC chromophilic	+	—
Patient 4—chromophobe/oncocytoma hybrid	++	+/−

++, strongly positive in a large percentage of the tumour; +, positive, +/−, focal areas of positivity.

^*^Denotes tumours from the same patient.

^†^Denotes tumours from the same patient.
