# Supplementary material for: Negative regulation of EGFR signalling by the human folliculin tumour suppressor protein
Source: Nat Commun. 2017 Jun 28;8:15866. doi: 10.1038/ncomms15866 (PMC5493755; doi:10.1038/ncomms15866)
Supplement: Supplementary Information [file ncomms15866-s1.pdf]

Type of file: pdf
Title of file for HTML: Supplementary Information
Description: Supplementary Figures, Supplementary Table and Supplementary References

Type of file: xlsx
Title of file for HTML: Supplementary Data 1
Description: Spectral counts from the mass spectrometry analysis of UOK257 cells following FLCN immunoprecipitation.

Supplementary Table 1. List of *Schizosaccharomyces pombe* strains used in this study.

Strain	Genotype	Source
12	h^+ *leu1-32 ade6-216 ura4-D18 imr1*R*(NcoI)::*$ura4^+$ *oriI*	1
565	h^+ *leu1-32 ade6-216 ura4-D18 imr1*R*(NcoI)::*$ura4^+$ *oriI bhd1*Δ*::* hph^R	2
699	h^+ *leu1-32 ade6-216 ura4-D18 imr1*R*(NcoI)*::$ura4^+$ *oriI ypt71*Δ*::* nat^R	2
729	h^+ *leu1-32 ade6-216 ura4-D18 imr1*R*(NcoI)*::$ura4^+$ *oriI ypt7*Δ*::* nat^R	2
736	h^+ *leu1-32 ade6-216 ura4-D18 imr1*R*(NcoI)::*$ura4^+$ *oriI bhd1*Δ*::* hph^R *ypt7*Δ*::* nat^R	2
766	h^+ *leu1-32 ade6-216 ura4-D18 imr1*R*(NcoI)::*$ura4^+$ *oriI bhd1*Δ*::* hph^R *ypt71*Δ*::* nat^R	2
769	h^- *leu1-32 ade6-216 ura4-D18 imr1*R*(NcoI)::*$ura4^+$ *oriI bhd1*Δ*::* hph^R	2
773	h^- *leu1-32 ade6-216 ura4-D18 imr1*R*(NcoI)*::$ura4^+$ *oriI ypt7*Δ*::* nat^R	2
779	h^- *leu1-32 ade6-216 ura4-D18 imr1*R*(NcoI)::*$ura4^+$ *oriI*	2
780	h^- *leu1-32 ade6-216 ura4-D18 imr1*R*(NcoI)*::$ura4^+$ *oriI ypt71*Δ*::* nat^R	2
FY17944	*ura4D-18 leu 1-32 ypt7Δ::ura4+ ypt71Δ::ura4+*	3

1 = [1]; 2 = *This study* 3 = Published in [2], obtained from YGRC, Yeast Genetic Resource Center Japan (http://yeast.lab.nig.ac.jp/nig/).

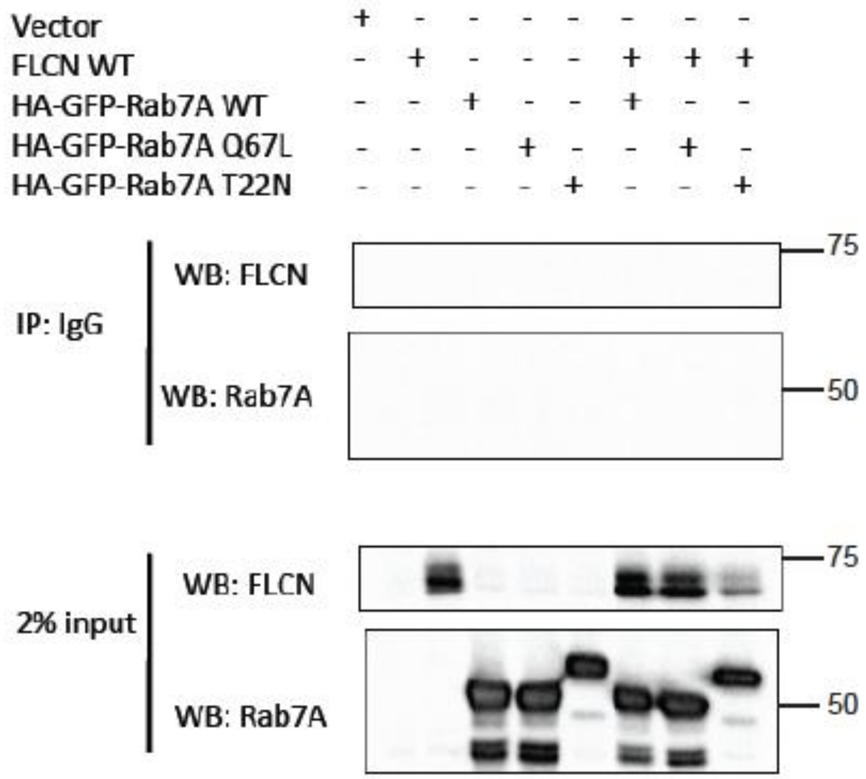

Supplementary Figure 1. IgG control immunoprecipitation for Figure 1A demonstrating that FLCN and Rab7A do not bind the Rabbit IgG non-specifically.

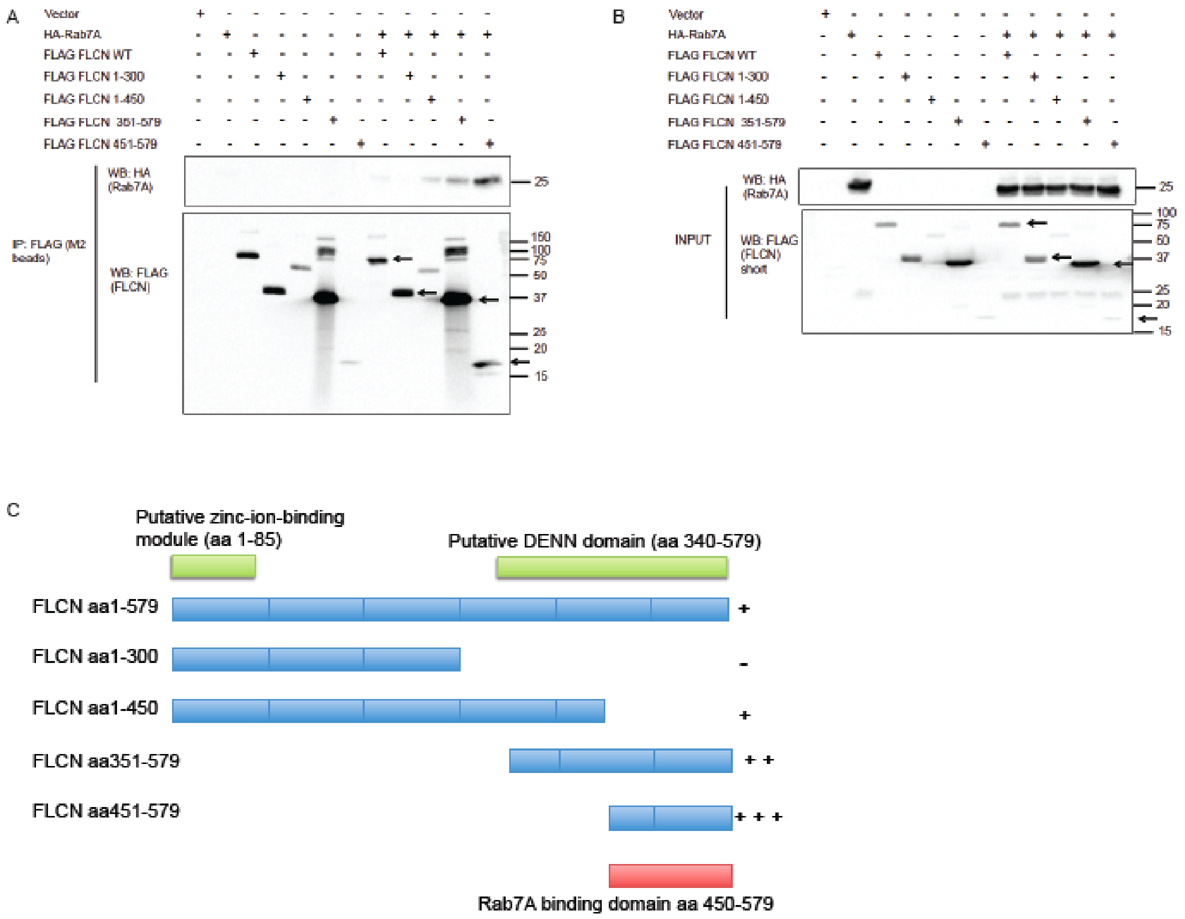

Supplementary Figure 2. A) The C terminus of FLCN binds strongly to Rab7A. 293T cells were co-transfected with HA-Rab7A (N terminus tag) and a FLAG-FLCN (N terminus tag) wild type (WT) or N- and C-terminus truncation mutants. Co-immunoprecipitation of Rab7A and various FLAG-FLCN truncation mutations demonstrates that the C terminus of FLCN (amino acids 451-579) binds strongly to Rab7A. B) Immunoblot demonstrating input expression levels of Rab7A and FLCN for each of the co-IPs in Supplementary Figure 2A. C) Schematic demonstrating that the Rab7A binding domain of FLCN is in the C-terminus (amino acids 450-579).

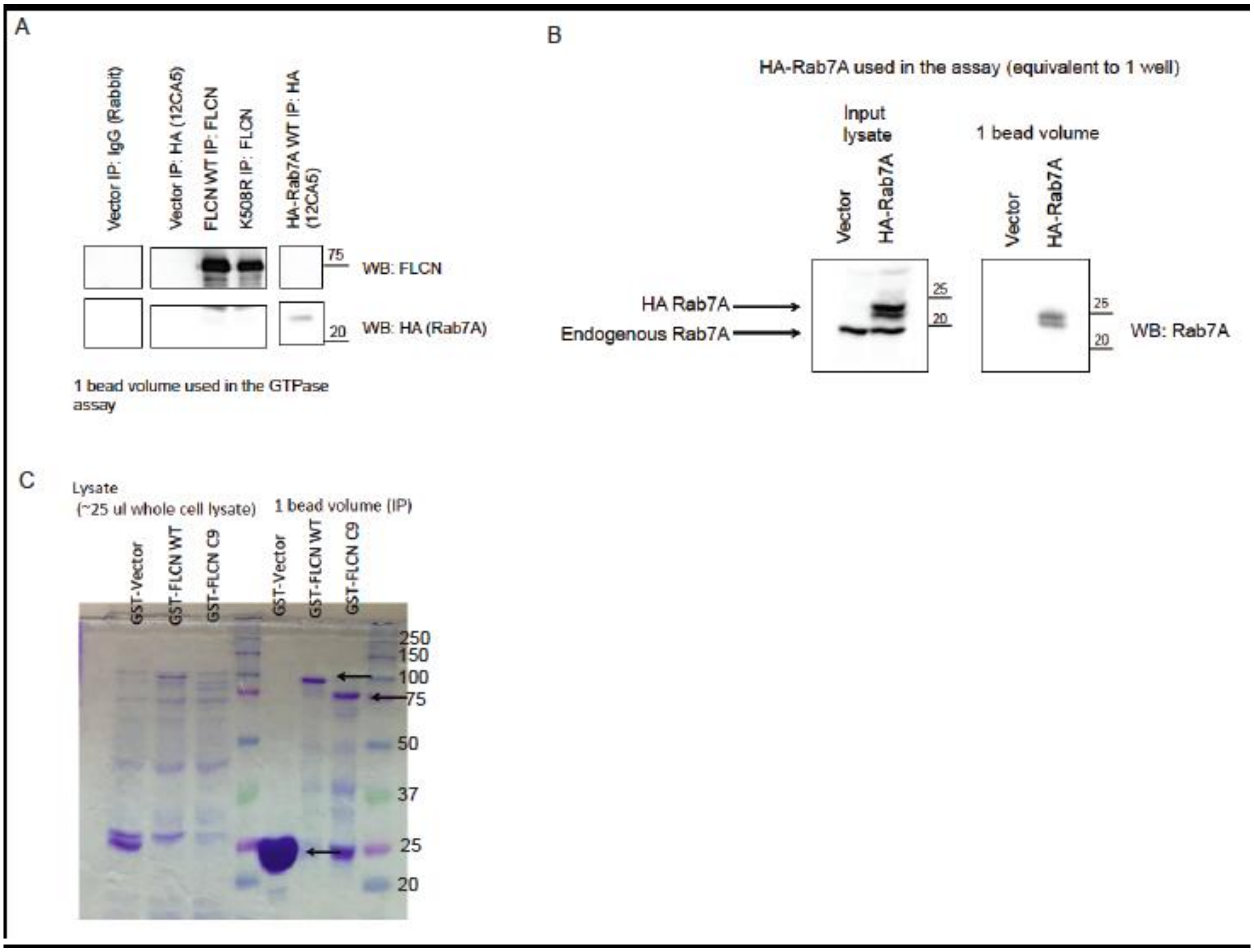

Supplementary Figure 3. A) For each GTPase assay, protein was freshly purified and immunoprecipitated and the beads were then equally distributed into each well of the assay. A Western blot confirmed that the FLCN WT, FLCN K508R and Rab7A WT proteins were expressed and purified. The signal represents the amount of purified protein in one bead volume, which is equivalent to the amount of purified protein in each well of the assay. This data represents one replicate of the assays in Figure 1E. B) The "input" lanes demonstrate that HA-Rab7A protein was expressed in transiently transfected 293T cells, and the "1 bead volume" lanes show the amount of purified Rab7A protein in one replicate of the assays in Figure 1F. For each replicate of the GTPase assay, protein was freshly purified and immunoprecipitated and the beads were then equally distributed into each well of the assay. C) Coomassie stained proteins following a GST purification of the GST-Vector (pGEX4T3), GST-FLCN WT, or GST-FLCN C9 mutant from BL21 bacteria cells. The signal represents the amount of purified protein in one bead volume, which was run in each well of the assay. This data represents one replicate from the assays in Figure 1F.

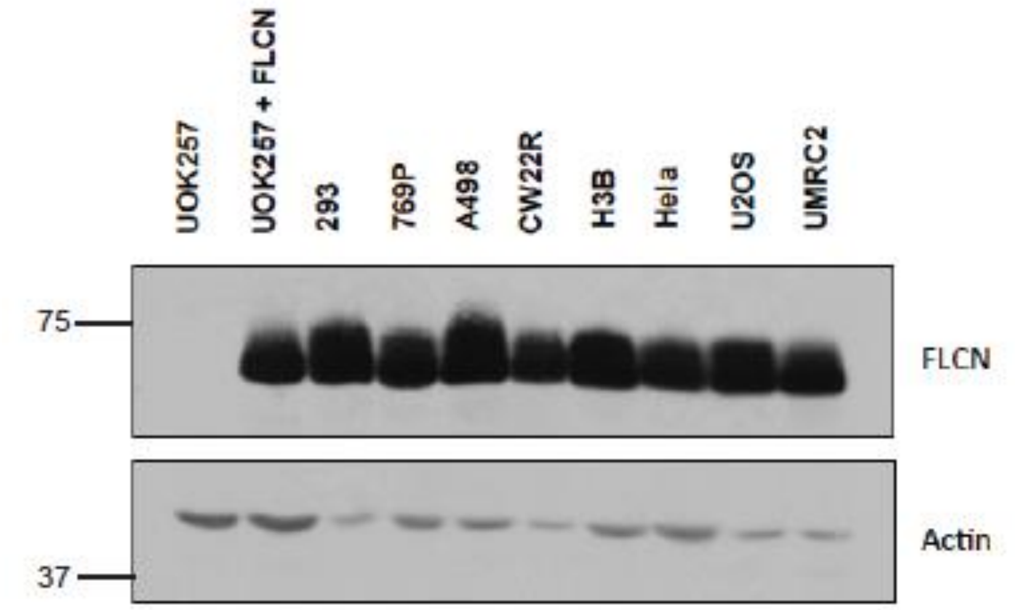

Supplementary Figure 4. Expression of FLCN in several cancer cell lines, including the FLCN deficient UOK257 cells and their isogenic controls replete with FLCN WT. Actin was used as a loading control.

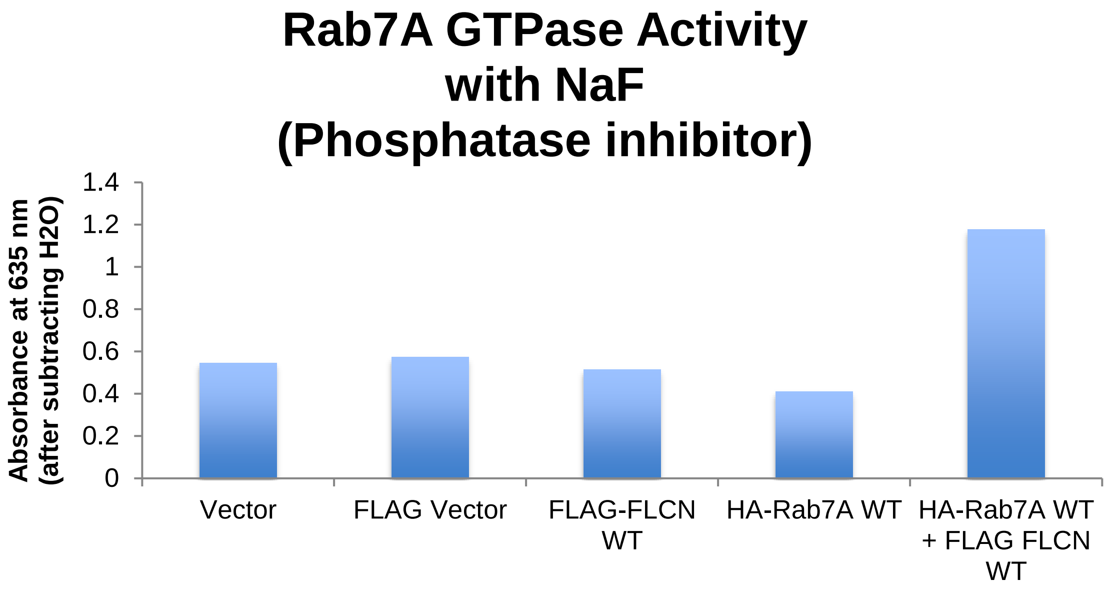

Supplementary Figure 5. FLAG-FLCN WT increases the GTPase activity of Rab7A even in the presence of the phosphatase inhibitor NaF. HA-Rab7A and FLAG-tagged FLCN WT proteins were purified from transfected 293T cells by immunoprecipitation with anti-HA antibodies (Rab7A) or anti-FLAG antibodies (Anti-FLAG M2 affinity gel). Purified HA-Rab7A and FLAG-FLCN WT proteins were incubated with the GTP substrate and a phosphatase inhibitor (NaF) at 37°C for 3 hours. The amount of inorganic phosphate released due to hydrolysis of GTP was measured using a commercially available GTPase colorimetric assay kit. Even in the absence of phosphatases, FLCN WT increased Rab7A's hydrolysis of GTP.

Supplementary Figure 6. Full western blot images from the most important blots. The images are merged images of the chemiluminescent image over the white light image showing the PVDF membrane and molecular weight markers (images taken with the Bio-Rad ChemiDoc system and the BioRad Image Lab Software (Bio-Rad Laboratories, Hercules, CA)). Only one exposure (generally the longest exposed image) is presented.

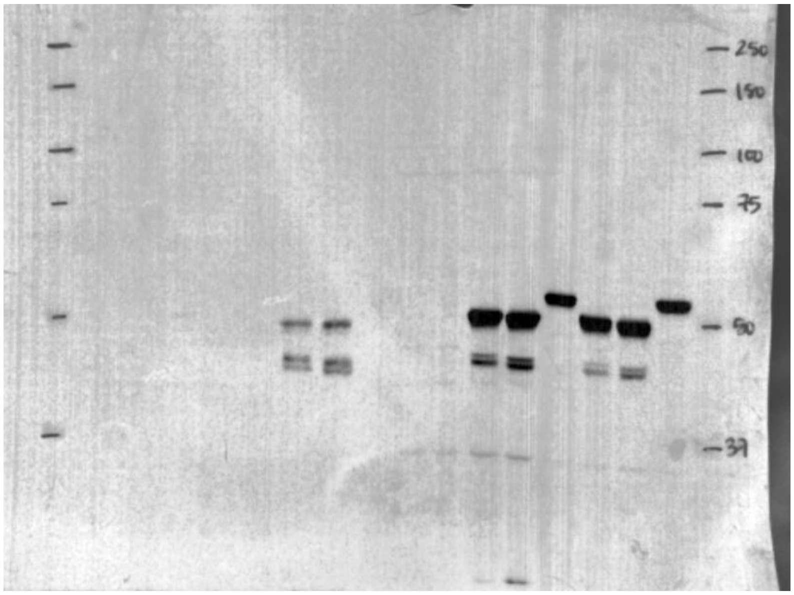

Full blot. Figure 1A. IP: FLCN. WB: Rab7A (left, IP lanes; right, input lanes).

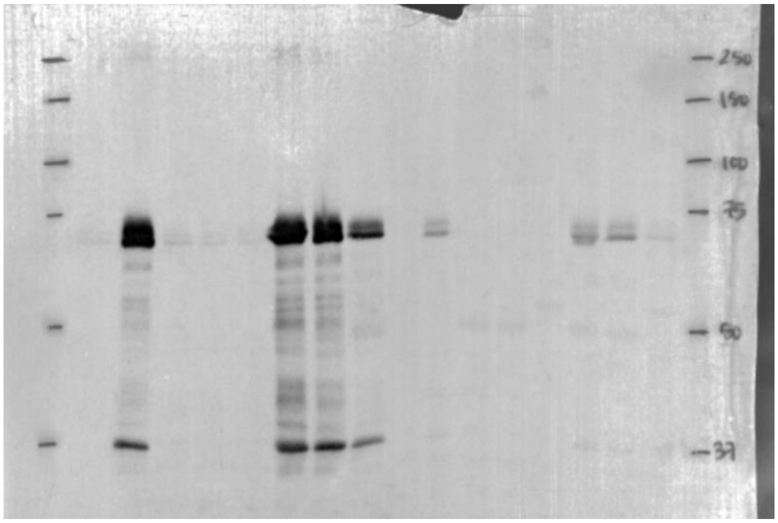

Full blot. Figure 1A. IP: FLCN. WB: FLCN (left, IP lanes; right, input lanes).

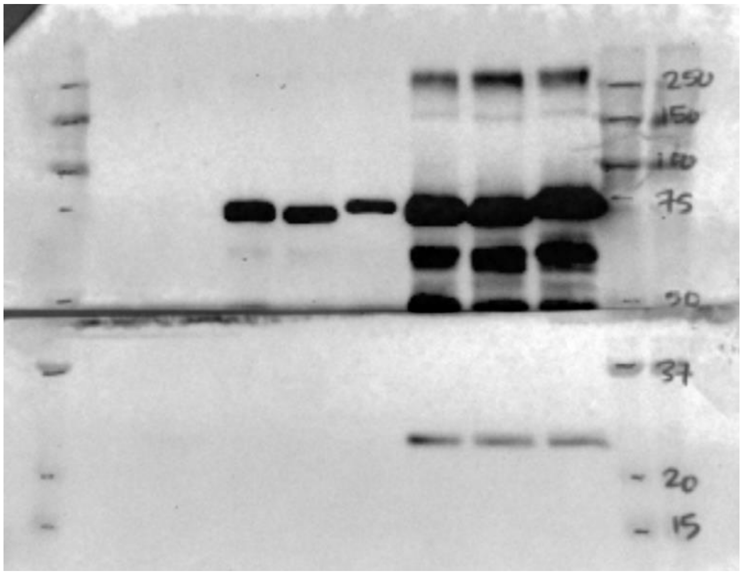

Full blot. Figure 1B. IP: FLAG. WB: FLCN, HA (Rab7A).

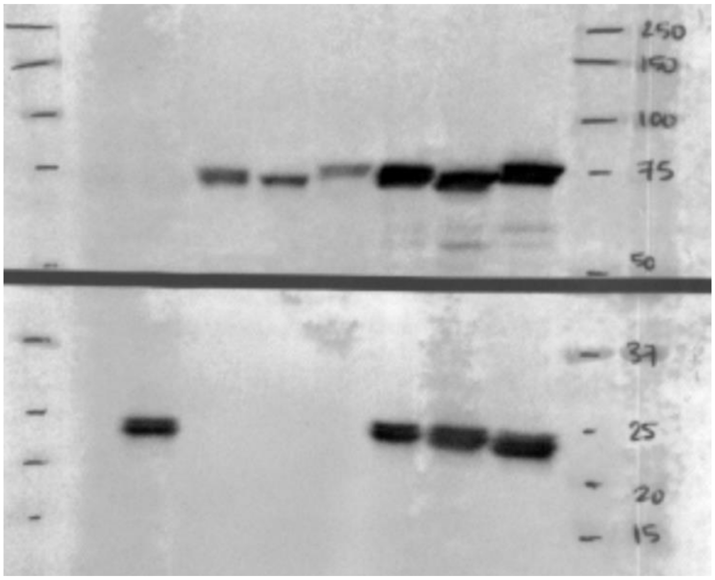

Full blot. Figure 1B. Input. WB: FLCN, HA (Rab7A).

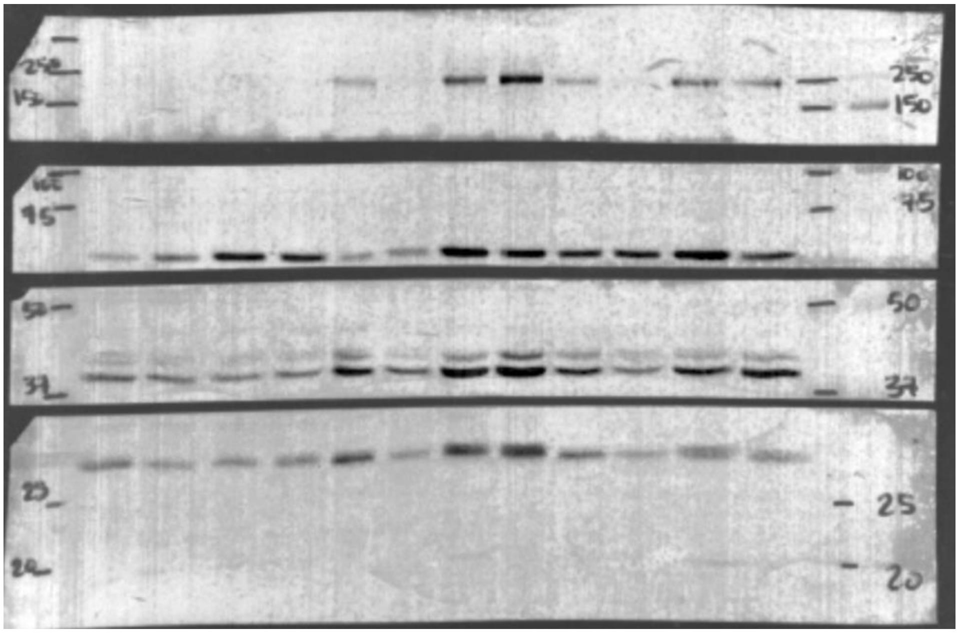

Full blot. Figure 3A. WB: pEGFR, pERK and pS6.

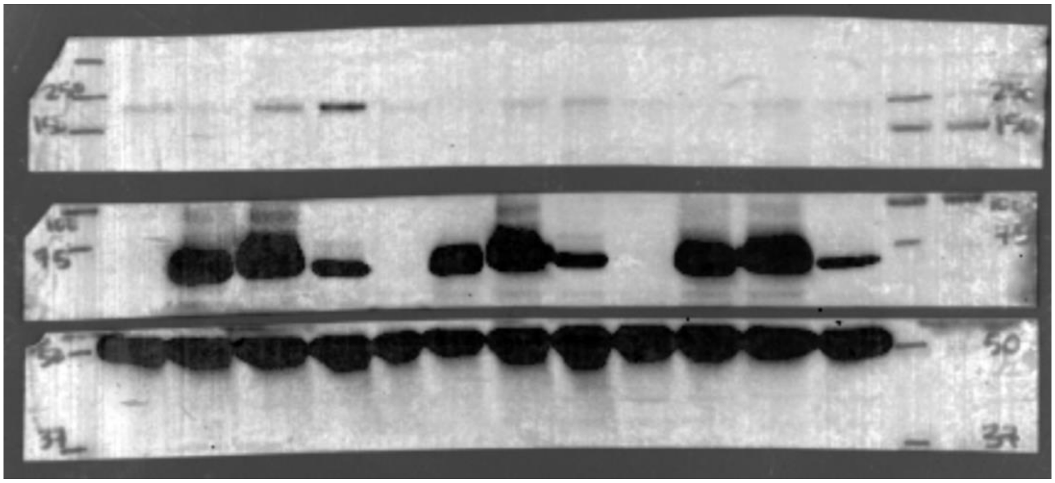

Full blot. Figure 3A. WB: Total EGFR, FLCN, Tubulin

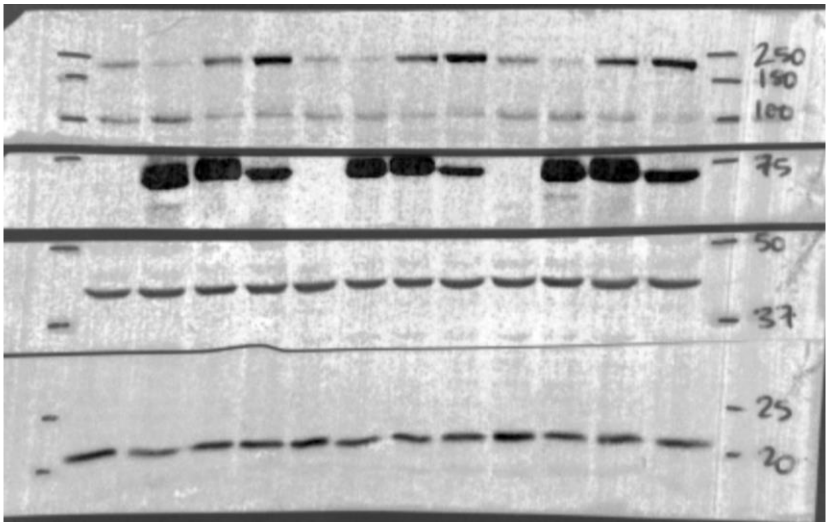

Full blot. Figure 3C. WB: Total EGFR, FLCN, Actin, Rab7A.

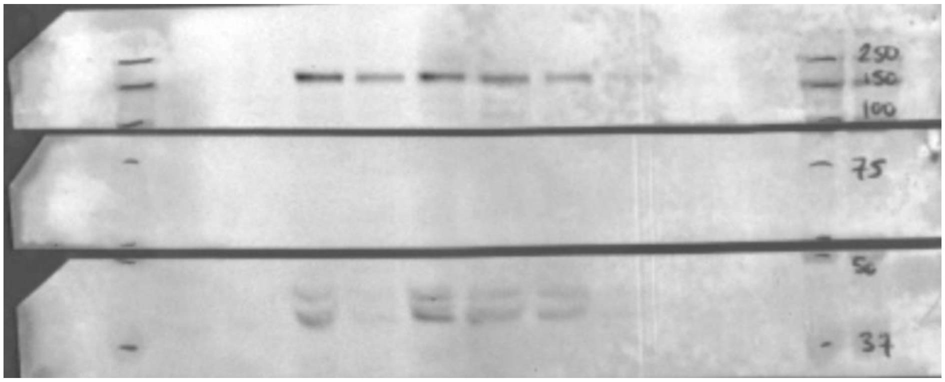

Full blot. Figure 3E. WB: pMET, pERK.

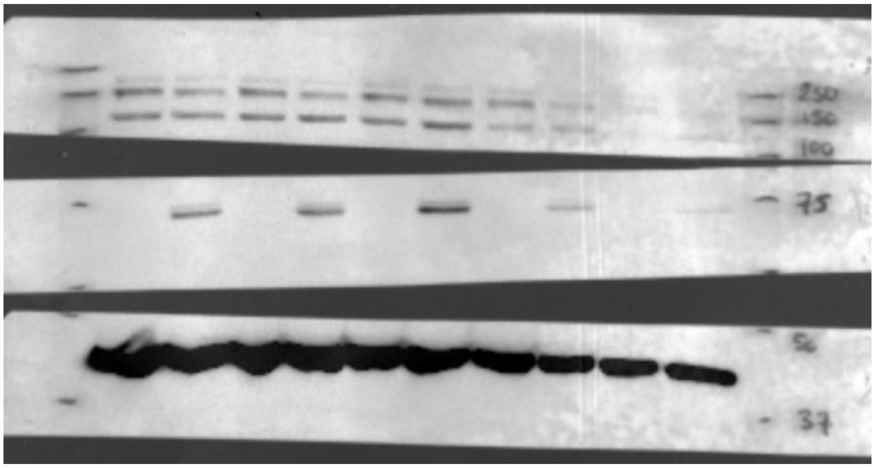

Full blot. Figure 3E. WB: Total MET, FLCN, Actin

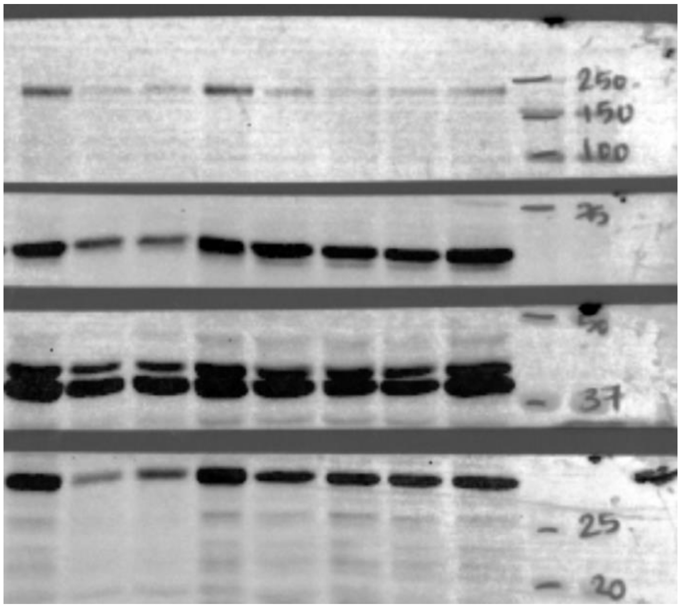

Full blot. Figure 4A. WB: pEGFR, pERK, pS6

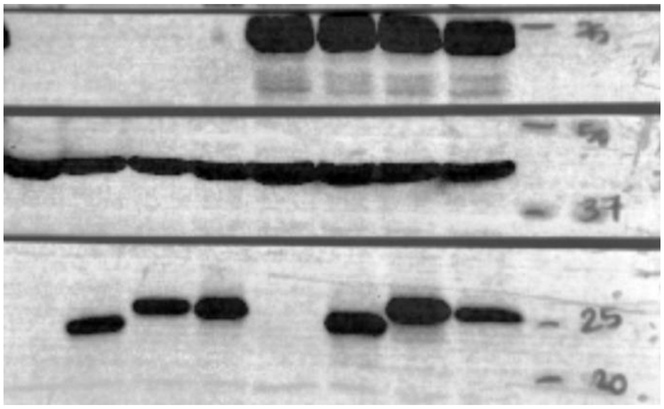

Full blot. Figure 4A. WB: FLCN, Actin, HA (Rab7A)

Supplementary References

1 Motamedi, M. R. *et al.* Two RNAi complexes, RITS and RDRC, physically interact and localize to noncoding centromeric RNAs. *Cell* **119**, 789-802, doi:10.1016/j.cell.2004.11.034 (2004).

2 Kashiwazaki, J., Iwaki, T., Takegawa, K., Shimoda, C. & Nakamura, T. Two fission yeast rab7 homologs, ypt7 and ypt71, play antagonistic roles in the regulation of vacuolar morphology. *Traffic* **10**, 912-924, doi:10.1111/j.1600-0854.2009.00907.x (2009).
